# Targeting epigenetic regulators for inflammation: Mechanisms and intervention therapy

**DOI:** 10.1002/mco2.173

**Published:** 2022-09-15

**Authors:** Su Zhang, Yang Meng, Lian Zhou, Lei Qiu, Heping Wang, Dan Su, Bo Zhang, Kui‐Ming Chan, Junhong Han

**Affiliations:** ^1^ Laboratory of Cancer Epigenetics and Genomics Frontiers Science Center for Disease‐Related Molecular Network State Key Laboratory of Biotherapy West China Hospital Sichuan University Chengdu China; ^2^ Department of Neurosurgery Tongji Hospital of Tongji Medical College Huazhong University of Science and Technology Wuhan China; ^3^ Laboratory of Cancer Epigenetics and Genomics Department of Gastrointestinal Surgery Frontiers Science Center for Disease‐Related Molecular Network West China Hospital Sichuan University Chengdu China; ^4^ Department of Biomedical Sciences City University of Hong Kong Hong Kong China

**Keywords:** epigenetic regulator, immune, inflammation, posttranslational modification (PTM)

## Abstract

Emerging evidence indicates that resolution of inflammation is a critical and dynamic endogenous process for host tissues defending against external invasive pathogens or internal tissue injury. It has long been known that autoimmune diseases and chronic inflammatory disorders are characterized by dysregulated immune responses, leading to excessive and uncontrol tissue inflammation. The dysregulation of epigenetic alterations including DNA methylation, posttranslational modifications to histone proteins, and noncoding RNA expression has been implicated in a host of inflammatory disorders and the immune system. The inflammatory response is considered as a critical trigger of epigenetic alterations that in turn intercede inflammatory actions. Thus, understanding the molecular mechanism that dictates the outcome of targeting epigenetic regulators for inflammatory disease is required for inflammation resolution. In this article, we elucidate the critical role of the nuclear factor‐κB signaling pathway, JAK/STAT signaling pathway, and the NLRP3 inflammasome in chronic inflammatory diseases. And we formulate the relationship between inflammation, coronavirus disease 2019, and human cancers. Additionally, we review the mechanism of epigenetic modifications involved in inflammation and innate immune cells. All that matters is that we propose and discuss the rejuvenation potential of interventions that target epigenetic regulators and regulatory mechanisms for chronic inflammation‐associated diseases to improve therapeutic outcomes.

## Graphical abstract

Innate immune system responds to stimulus (bacteria, virus, lipopolysaccharide) firstly; nevertheless, exaggerated immune response results in a variety of inflammation‐related diseases. Epigenetic modifications including chromatin remodeling, DNA modifications, RNA modifications, Histone modifications, noncoding RNA, and regulate multiple signaling pathways of chemokines and cytokines released by immune cells in inflammation response. Biotherapeutics targeting epigenetics regulators have been studied as an important clinically effective treatments for inflammation‐related diseases.

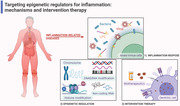

## 1 INTRODUCTION

Inflammation is a physiological process mounted by the immune system following pathogen and inflammatory cytokine stimulation and is crucial for host protection from invasive pathogens.^1^, ^2^ Upon sensing stimulation, innate immune cells, including neutrophils and macrophages, are recruited and recognized invading pathogens or cell damage with germline‐encoded pattern recognition receptors (PRRs), examples including Toll‐like receptors (TLRs), resulting in the activation of both innate and adaptive immune responses.^1^, ^3^ Concomitantly, these cells phagocytose pathogens and secrete proinflammatory cytokines, including cytokines, autoantibodies, efferocytosis, prostanoids, and matrix metalloproteinases, activating downstream proinflammatory signaling pathways.^4^, ^5^ Of note, uncontrolled inflammation is the primary cause of a myriad of chronic inflammatory diseases, which consist of rheumatoid arthritis (RA),^6^ cardiovascular diseases,^7^ systemic lupus erythematosus (SLE),^8^ and neurodegenerative diseases (ND),^9^ even resulting in host death during pathogen infection.^10^, ^11^ As a recent example of virus‐induced severe acute respiratory syndrome coronavirus 2 (SARS‐CoV‐2) infection, it is involved into defective type I interferon (IFN) activity and cytokine storms characterized by high levels of interleukin (IL)‐1β, IL‐6, IL‐12, and tumor necrosis factor (TNF), subsequently leading to acute respiratory distress syndrome (ARDS).^12^, ^13^

Epigenetics aims to the study of mechanisms that are heritable and reversible alternations in histone or DNA modifications being responsible for gene expression without changing the primary DNA sequence.^14^, ^15^ Dysregulation of epigenetic modifications that disrupt cellular gene expression patterns leads to complex diseases, including autoimmune diseases,^16^ cancers,^17^ diabetes,^18^ and neurological disorders,^19^ and occurs in several other maladies. These related diseases have been extensively reviewed.^20^ Most recently, extensive researches on aberrant epigenetic modifications in autoimmune diseases and inflammation were conducted and attracted significant interest. Mounting evidence suggests that epigenetic regulation is an essential driver involved in inflammatory diseases.^21^, ^22^, ^23^ Nevertheless, the epigenetic mechanisms underlying the different disease phenotypes remain elusive and represent an intense area of investigation.^24^

**FIGURE 1:**
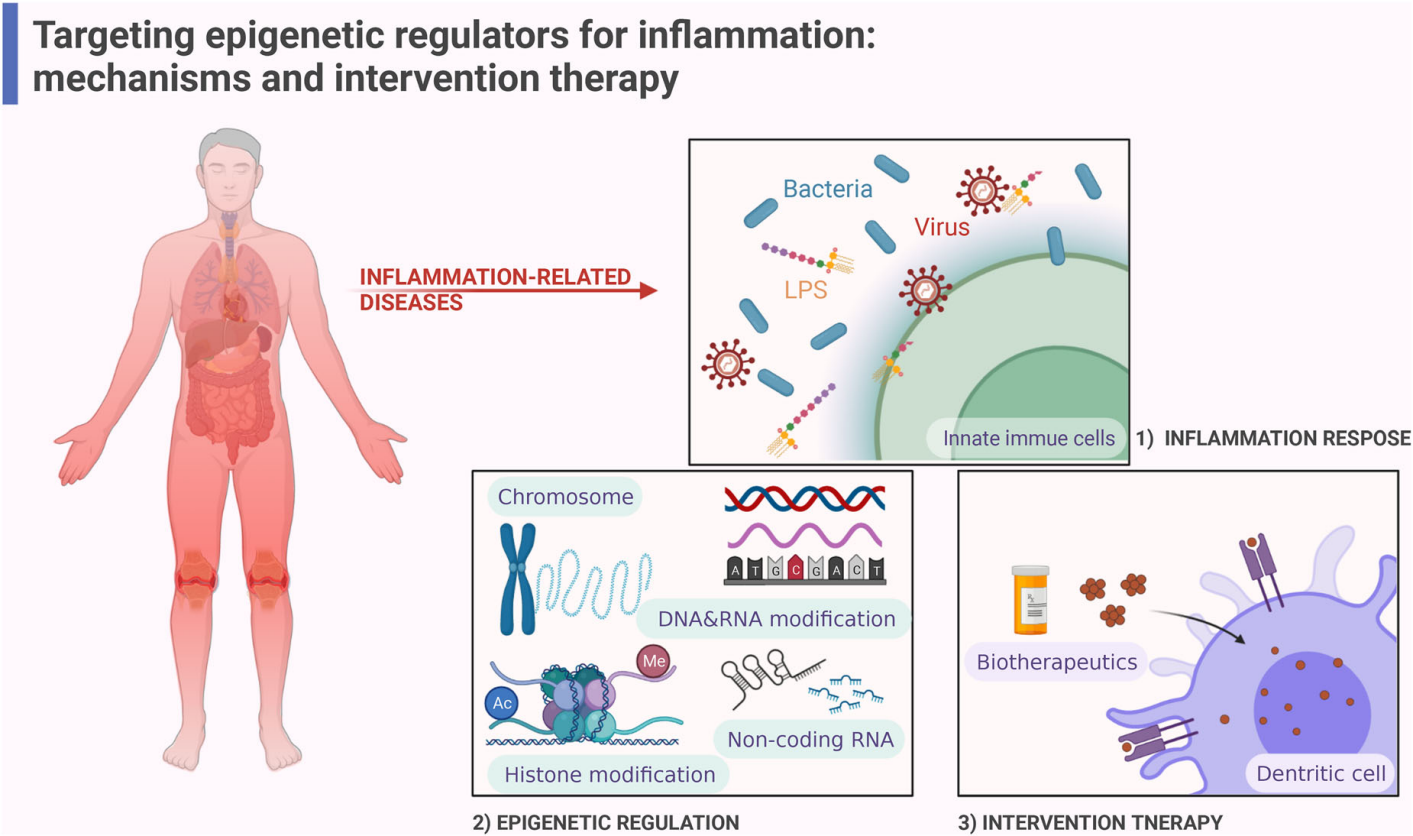
Intervention therapy of epigenetic regulators targeting inflammation‐related diseases. Innate immune system responds to stimulus (bacteria, virus, lipopolysaccharide [LPS]) firstly, nevertheless exaggerated immune response results in a variety of inflammation‐related diseases. Epigenetic modifications including chromatin remodeling, DNA modifications, RNA modifications, Histone modifications, noncoding RNA, regulate multiple signaling pathways of chemokines, and cytokines released by immune cells in inflammation response. Biotherapeutics targeting epigenetics regulators have been studied as an important clinically effective treatments for inflammation‐related diseases (created with BioRender.com).

The already available mechanistic insights into the regulation of epigenetic modification, including DNA methylation, histone posttranslational modifications (PTMs), RNA modification, microRNA (miRNA) and long noncoding RNA (lncRNA), suggesting vital roles in the adjustment of inflammation and innate immune cells. This review mainly focuses on the inflammation‐related epigenetic mechanisms and summarizes the known contributions of targeting epigenetic modification to therapy in inflammation‐related diseases (Figure 1).

## 2 INFLAMMATION‐RELATED DISEASES

### 2.1 Definition of inflammation

Inflammation is the immediate response of the host tissues and cells to pathogens, harmful stimuli (such as chemicals), or physical damage. The innate immune system responds rapidly to inflammation, which mainly comprises inducers, sensors, mediators, and effectors.^25^ PRRs act as surface receptors of immune cells, including the family of TLRs, C‐type lectin, RIG‐I‐like, and cytosolic PRRs like Nod‐like receptors. These receptors recognize damage‐associated molecular patterns and pathogen‐associated molecular patterns (PAMPs).^26^ The recognition of such molecular patterns sets off the synthesis of inflammatory mediators, which are classified into seven groups based on their biochemical properties, including cytokines, chemokines, vasoactive amines, vasoactive peptides, fragments of complement components, eicosanoids, and proteolytic enzymes.^25^ Inflammatory mediators act on target tissues to recruit monocytes and neutrophils from the activated endothelium of blood vessels, and activate of macrophages in host defense.^27^

**FIGURE 2:**
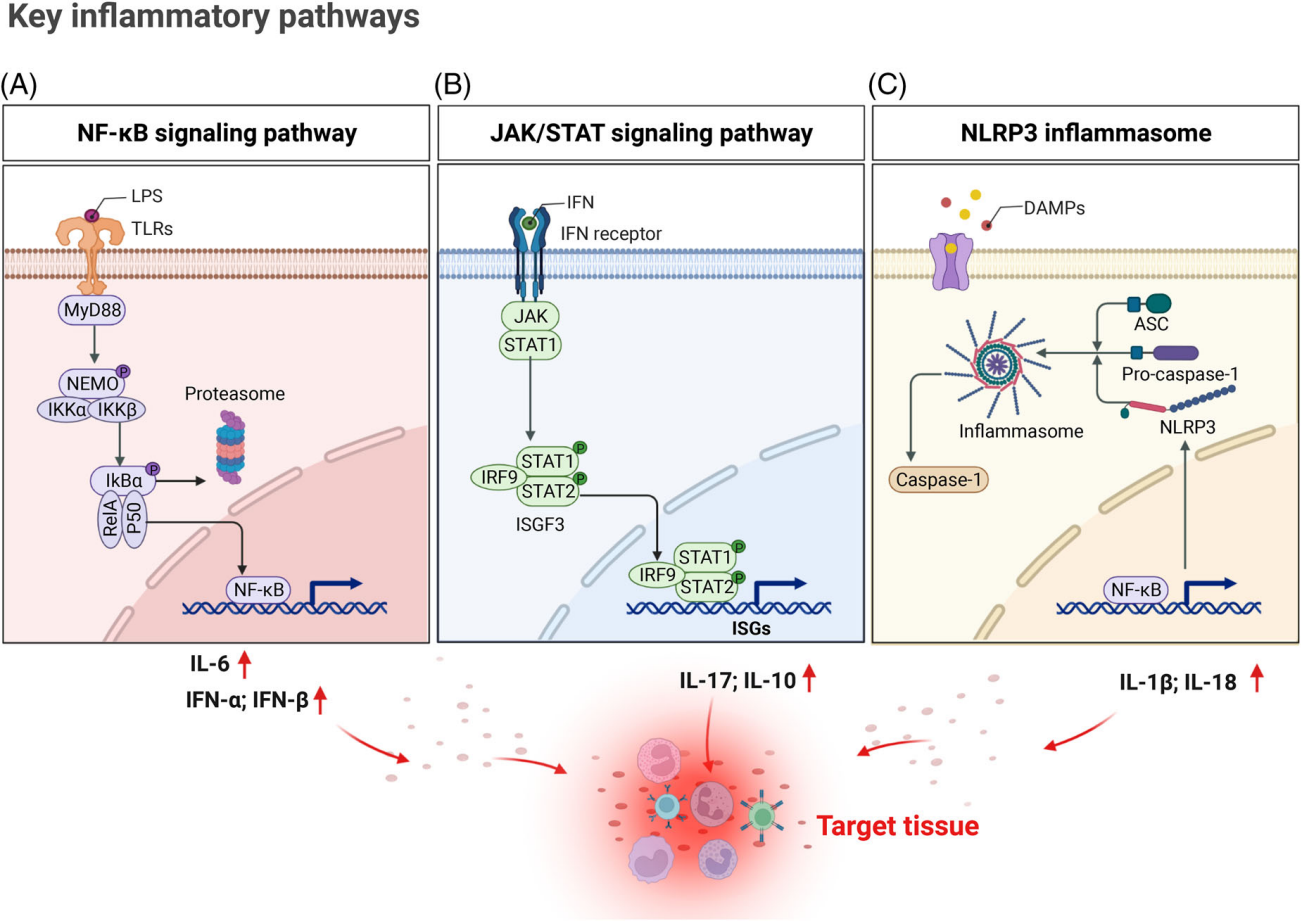
Activation of key signaling pathways in immune cells during inflammation response. Signal (A): In the canonical nuclear factor‐κB (NF‐κB) signaling pathway, lipopolysaccharide (LPS) activates Toll‐like receptors (TLRs), and MyD88 delivers this signaling to a serious of adaptor protein leading to activation of IκB kinase (IKK)β in IKK complex, subsequently activating phosphorylation of IκBα, which is degraded by the proteasome in further. NF‐κB (RelA and P50) homo‐ or heterodimers translocated into nucleus to regulate target genes expression, such as interleukin (IL)‐6, interferons (IFNs). Signal (B): Type Ⅰ IFN activate canonical signaling pathways leading JAK/STAT cascades signaling transduction. ISGF3 complex is formed by IRF9 and phosphorylation STAT1 STAT2, activating downstream targeting genes interferon‐stimulated genes (ISGs) (examples, IL‐17, IL‐10) after transporting into the nucleus. Signal (C): In the canonical NLRP3 (Nod‐like receptor family pyrin domain containing 3) inflammasome pathway, expression of NLRP3 is regulated by MyD88–NF‐κB pathway. Once activation, NLRP3 undergoes oligomerization, recruit ASC (apoptosis‐associated speck‐like protein containing CARD) and pro‐caspase‐1 to assemble NLRP3 inflammasome, which induces caspase activation and IL‐1β, IL‐18 maturation. These cytokines promote inflammatory responses in target tissues (created with BioRender.com).

During an inflammatory response, various inflammatory signaling pathways are activated and organized to regulate the expression of both pro‐ and anti‐inflammatory mediators. Based on previous studies, nuclear factor‐κB (NF‐κB) has been regard a prototypical proinflammatory signaling pathway^28^ (Figure 2). In the canonical pathway, TLRs and proinflammatory cytokines, such as TNF‐α and IL‐1 stimulate excitatory signaling, leading to the activation of the IκB kinase (IKK) complex, which then phosphorylate IκBs. Subsequently, NF‐κB subunits (p50/RelA) translocate into nucleus and activate the target gene expression. In the alternative NF‐κB pathway, B‐cell activation factor, lymphotoxin β‐receptor, CD40 L, and receptor activator for NF‐κB (RANKL) activate IKKα to phosphorylates p100 leading to the processing of p100 into p52 and the nuclear translocation of p52/RelB. Eventually, p52/RelB complex initiates the expression of target genes.^28^, ^29^

Elevated production of IFNs during inflammatory response leads to upregulation of most typically canonical interferon‐stimulated genes (ISGs) expression. IFNs are composed of type I IFNs and the sole type II IFN (IFN‐γ) (Figure 2), which have been implicated in infection and autoimmune diseases by binding specific cell‐surface receptors expressed on most cell types. IFNs activate target gene expression through the signaling pathway of protein tyrosine kinases JAKs and STATs. Type I IFNs interact with their heterodimeric receptor IFNAR, and motivate the receptor‐associated protein tyrosine kinases JAK1 and TYK2, which activate phosphorylation of STAT1 and STAT2, following their combination with the transcription factor IRF9. The heterotrimeric complex ISGF3 activate ISGs, including genes encoding antiviral proteins and various transcription factors like interferon‐regulatory factors (IRFs).^30^ IFN‐γ (Figure 2) activates JAK1, JAK2, and predominantly STAT1 homodimers,^31^ which bind a distinct DNA element called a γ activated site and directly activate a diverse set of ISGs, such as CXCL10 and IRFs.^32^

Nod‐like receptor family pyrin domain containing 3 (NLRP3) is an intracellular receptor that senses foreign pathogens and endogenous danger signals, conducting the formation and activation of the NLRP3 inflammasome (Figure 2), which is closely associated with innate immunity and inflammation.^33^ Once activated, NLRP3 recruits an adaptor (an apoptosis‐related spot‐like protein containing CARD domain [ASC]) and an effector (caspase‐1) to form NLRP3 inflammasome, which assembles and activates caspase‐1 (Figure 2). This signaling pathway induces pyroptosis and cleaves proinflammatory cytokines IL‐1β and IL‐18 into active forms, promoting the development of inflammatory responses.^34^

Under normal circumstances, inflammation quickly ends after the clearance of infection and injurious agents. There is precise control of the complex networks of inflammatory pathways to limit tissue damage during inflammation, while continued activation of the immune system can lead to inflammatory dysregulation. Growing evidence suggests a close link between inflammation and many chronic health conditions, including autoimmune diseases, ND, viral infections such as coronavirus disease 2019 (COVID‐19), and cancer.^35^

### 2.2 Chronic inflammatory diseases

Chronic inflammatory diseases make up a burden of human health. Although the origins of some chronic diseases remain to be clarified, the dysregulated inflammatory response has been evidenced to lead to pathological conditions.^5^ During infection, PPRs and cytokines continue to active innate immune cells through key inflammatory signaling pathways. Here, we discuss the relationship of dysregulated inflammatory signaling pathways to chronic inflammatory diseases, including SLE, RA, and ND.

#### 2.2.1 Systemic lupus erythematosus

SLE is a condition in which the immune system attacks healthy cells and tissues throughout the body. The pathogenesis of SLE is characterized by exaggerated immune responses and loss of tolerance against self‐antigens. Antibody production and defective elimination, complement and cytokine activation, and tissue deposition of immune complex lead to clinical manifestations ranging from mild fatigue and joint pain to severe, life‐threatening organ damage.^36^

The dysregulation of apoptosis and nuclear debris clearance contribute to an increase in self‐antigen exposure. Accumulated apoptotic debris can trigger TLRs and nucleic acid sensors that facilitate their clearance by cells in the reticuloendothelial compartment. TLRs, such as TLR3, TLR7, TLR8, and TLR9, are transferred into the endoplasmic reticulum by the trafficking protein unc‑93 homologue B1 (UNC93B1). TLR9 is a receptor for DNA containing unmethylated CpG sequence motifs. In plasmacytoid dendritic cells (DCs), TLR9 and IRF7 induce a strong IFN immune response in early endosomes, whereas TLR9 and NF‐κB drive a proinflammatory cytokine response in late endosomes.^37^ In addition, type I and type II IFNs are well‐established and play an important role in the pathogenesis of SLE. Studies have reported that transcripts of IFN‐α and ISGs could be detected in inflamed kidney and skin tissues of SLE patients,^38^ the JAK/STAT cascade pathway which was activated by IFNs, promoted the release of most cytokines associated with SLE pathogenesis and pain. Examples of cytokines and pro‐nociceptive factors that are regulated through the JAK/STAT member proteins include IFN expression (JAK1/STAT1/STAT2); IL‐4 secretion from B cells (JAK1/STAT2/STAT6); and IL‐6 and IL‐10 expressions (JAK1/STAT3).^39^

Accumulating evidence suggested that the NLRP3 inflammasome was hyper‐activated in SLE. In macrophages and peripheral blood mononuclear cells (PBMCs) of patients with SLE, dsDNA and anti‐dsDNA autoantibodies have been reported to upregulate NLRP3 and caspase‐1 expression, improving the production of IL‐1β.^40^, ^41^, ^42^ Additionally, Liu et al.^43^ found that prolonged IFN‐α exposure induced NLRP3 inflammasome activation and IL‐1β secretion through an IRF1 pathway in monocytes of SLE patients.

#### 2.2.2 Rheumatoid arthritis

RA is one of the most prevalent chronic inflammatory diseases that endanger human health, its predisposing factors mainly include positive family inheritance and environment factors, such as smoking and low socioeconomic status or educational attainment.^44^ Besides involving the joints, RA also induces a syndrome that includes extra articular manifestations, such as rheumatoid nodules, pulmonary involvement or vasculitis, and systemic comorbidities. Gene sequencing of RA revealed that more than a hundred loci were associated with disease risk and progression, prominent among these were the MHC class II locus, especially human leukocyte antigen DR01/04, implicating T cells recognizing autoreactive peptides.^45^

Early in the disease course of the RA, bone loss occurs due to continued direct exposure of bone and cartilage to the inflammatory microenvironment. This progress is driven by the induction of bone‐resorbing osteoclasts by autoantibodies, leading to the first structural changes in the pre‐disease stage and exacerbated by the action of proinflammatory cytokines.^44^ Osteoclasts, as the main bone‐resorbing cells, are highly sensitive to antibodies and inflammatory cytokines, especially TNF, IL‐1, and IL‐6/IL‐6R complex, all of which induce osteoclast differentiation either directly or indirectly through the NF‐κB ligand (RANKL). RANKL is involved in the inflammatory response and bone erosion as a ligand of NF‐κB.^46^ In 2018, Tanaka et al.^47^ called for research investigating RANKL as a new therapeutic target in RA. In addition, JAKs subserve the signaling pathways of many cytokine receptors in RA, including IL‐6, granulocyte‐macrophage colony‐stimulating factor, IFNs, and common g‐chain cytokines, such as IL‐7 and IL‐15.^45^ Recently, components of the NLRP3 inflammasome were found to be expressed in synovia of RA patients, and pentaxin 3 (PTX3), an essential component of innate immunity, has been suggested as a novel marker for the diagnosis of RA. Guo et al.^48^ and Wu et al.^49^ found that IL‐6 could drive PTX3 plus C1q‐induced NLRP3 over‐activation and pyroptosis in monocytes from RA patients.

#### 2.2.3 Neurodegenerative disease

Neuroinflammation initially protects the brain by removing or inhibiting diverse pathogens, promoting tissue repair and removing cellular debris. However, sustained inflammatory responses have a detrimental effect on health.^50^, ^51^, ^52^ Chronic inflammation of an innate immune response in the central nervous system (CNS) leads to neuronal damage, which is a principle hallmark of ND, such as Alzheimer's disease (AD) and Parkinson's disease (PD), amyotrophic lateral sclerosis.^9^

The CNS consists of two categories of cells: neurons and glial cells.^53^ Microglia (MG) are the main effectors of the CNS in inflammation response progression, and their function is like that of macrophages.^54^ MG responds quickly to harmful antigens and releases a variety of inflammatory factors, such as TNF‐α, IL‐6, nitric oxide.^55^ There are two states of MG activation, M1 pro‐inflammation phenotype and M2 anti‐inflammation phenotype. Lipopolysaccharide (LPS) and IFNs induce M1 activation through the NF‐κB pathway and JAK/STAT signaling, respectively.^56^ Astrocytes‐mediated reactive gliosis is also part of neuroinflammation. Inflammatory injury has been proposed to induce the “harmful” A1 astrocyte phenotype through the NF‐κB pathway, whereas ischemia induces the “protective” A2 phenotype via the STAT3 pathway.^57^

Amyloid‐β (Aβ) deposition in the brain is one of the starting events in AD. Aβ activates the NF‐κB pathway in astrocytes, leading to increased release of complement C3, which successively acts on C3a receptors on neurons and MG, resulting in neuronal dysfunction and microglial activation.^58^, ^59^ Cellular crosstalk between MG and astrocytes continues to be a positively regulation under the inflammatory milieu of AD, leading to further self‐amplification of inflammatory response. The recruitment of NLRP3 inflammasome has been a vital event in the proinflammatory response of MG, following accelerating the expression of caspase‐1 and maturation of IL‐1β.^60^, ^61^ The activation of NLRP3 inflammasome can also lead to the release of ASC, which might act as a binding core for Aβ aggregation.^57^, ^62^

### 2.3 Inflammation and COVID‐19

The COVID‐19 pandemic, caused by SARS‐CoV‐2, has spread worldwide.^63^ The pathophysiology of SARS‐CoV‐2 infection is similar to that of SARS‐CoV infection, with overbearing inflammatory responses and a strong association with the resulting damage to the airways.^64^ Severe COVID‐19 cases progress to ARDS on average roughly 8–9 days after symptom appear.^65^ Respiratory failure causes death in 70% of fatal COVID‐19 cases. Additionally, the massive release of cytokines by the immune system in response to viral infection and/or secondary infections can lead to a cytokine storm and symptoms of sepsis, which handle 28% of fatal COVID‐19 cases.^13^

For RNA viruses such as SARS‐CoV‐2, the innate immune response is initiated through the engagement of PRRs by viral single‐stranded RNA (ssRNA) and double‐stranded RNA (dsRNA). Upon PRR activation, downstream signaling cascades trigger the secretion of cytokines.^13^ Among these, TLRs upregulate the antiviral and proinflammatory mediators IL‐6 and IL‐8 and IFNs through the activation of NF‐κB.^66^ SARS‐CoV‐2 NSP9 and NSP10 might induce IL‐6 and IL‐8 production, potentially by inhibiting NF‐κB repressing factor, an endogenous NF‐κB repressor.^66^, ^67^ Collectively, these immune progresses induce imbalance between antiviral programs and proinflammatory response in target cells. In addition, Rodrigues et al.^68^ demonstrated that NLRP3 inflammasomes were activated in PBMCs and tissues of postmortem COVID‐19 patients upon autopsy. Inflammasome‐derived products such as Casp1p20 and IL‐18 in the sera correlated with IL‐6 and lactate dehydrogenase.

Similarly to SARS‐CoV, spike (S) protein is expressed on the surface of SARS‐CoV‐2 virus particles, and several receptors have been showed for the interaction of spike (S) protein with host cells, including angiotensin‐converting enzyme (ACE2). ACE2 has a crucial role because loss of pulmonary ACE2 function is associated with acute lung injury (ALI), and virus‐induced ACE2 downregulation may be important for disease pathology.^69^, ^70^

### 2.4 Inflammation, cellular senescence, and cancer

Inflammation predisposes patients to the development of cancer and facilitates all stages of tumorigenesis. Approximately 15%–20% of all cancer cases develop infection, chronic inflammation, or autoimmunity at the same tissue or organ site. Inflammation has a great impact on the composition of the tumor microenvironment (TME) and particularly on the plasticity of tumor cells, as well as surrounding stromal and inflammatory cells, engages in orchestrated interactions to shape the inflammatory TME.^71^, ^72^, ^73^

Several pathways are broadly implicated in inflammatory processes and are closely linked to tumorigenesis in multiple tissues. Part of the complexity and differences in NF‐κB function between different tumor types is because of its crosstalk with other transcription factors and signaling proteins. NF‐κB promotes STAT3‑mediated expression of genes encoding different inflammatory mediators in immune cells of the TME.^74^ In glioma stem cells, constitutive activation of NF‐κB and STAT3 promotes upregulation of Notch signaling pathway.^74^ Elevated levels of STAT3 is observed in tumor‐infiltrating immune cells and plays negative regulatory effects on neutrophils, natural killer (NK) cells, effector T cells, and DCs, indicating that STAT3 activation in immune cells likely leads to the down‐modulation of antitumor immunity.^75^ Furthermore, the NLRP3 inflammasome has been shown closely associated with the development of several cancers. In breast cancer, the NLRP3 inflammasome and IL‐1β production induce the infiltration of myeloid cells, such as tumor‐associated macrophages (TAMs) and myeloid‐derived suppressor cells, forming an inflammatory microenvironment and thus promoting breast cancer progression.^76^

While cellular senescence intrinsically suppresses the tumorigenesis of preneoplastic cells, senescent cells show widespread changes in gene expression and chromatin organization. These changes include the secretion of numerous proinflammatory cytokines, chemokines, growth factors, and proteases, which is a feature known as the senescence‐associated secretory phenotype (SASP). The SASP produced by senescent cells can actually extrinsically promote tumor growth, relapse, and metastasis.^77^, ^78^ Most SASP components are regulated by NF‐κB, STAT3, CEBP/β, and mTOR. Indeed, inhibition of the JAK pathway leads to reprogramming of the SASP, eliminating the negative components of these factors. Recent data investigated that the SASP could be controlled by the cGAS/STING pathway. cGAS, a DNA sensor, triggers cellular senescence and controls gene transcription of SASPs through the adaptor protein STING.^79^, ^80^ Overall, these results demonstrate senescent cells can detrimentally contribute to tumor progression by influencing the inflammatory response via paracrine signaling.^81^

## 3 EPIGENETIC ROLES IN REGULATING INFLAMMATORY DISEASE

### 3.1 Chromatin remodeling and inflammation

Chromatin structure dynamics is necessary for regulating gene expression, and is mostly controlled by a group of ATP‐dependent chromatin remodeling regulators which have an ATPase domain of catalytic subunits. ATP‐dependent chromatin remodeling regulators use energy from ATP hydrolysis to modulate DNA translocation on nucleosome and histone composition, regulate the assembly, disassembly, and rearrangement of nucleosomes on chromatin.^82^, ^83^

**FIGURE 3:**
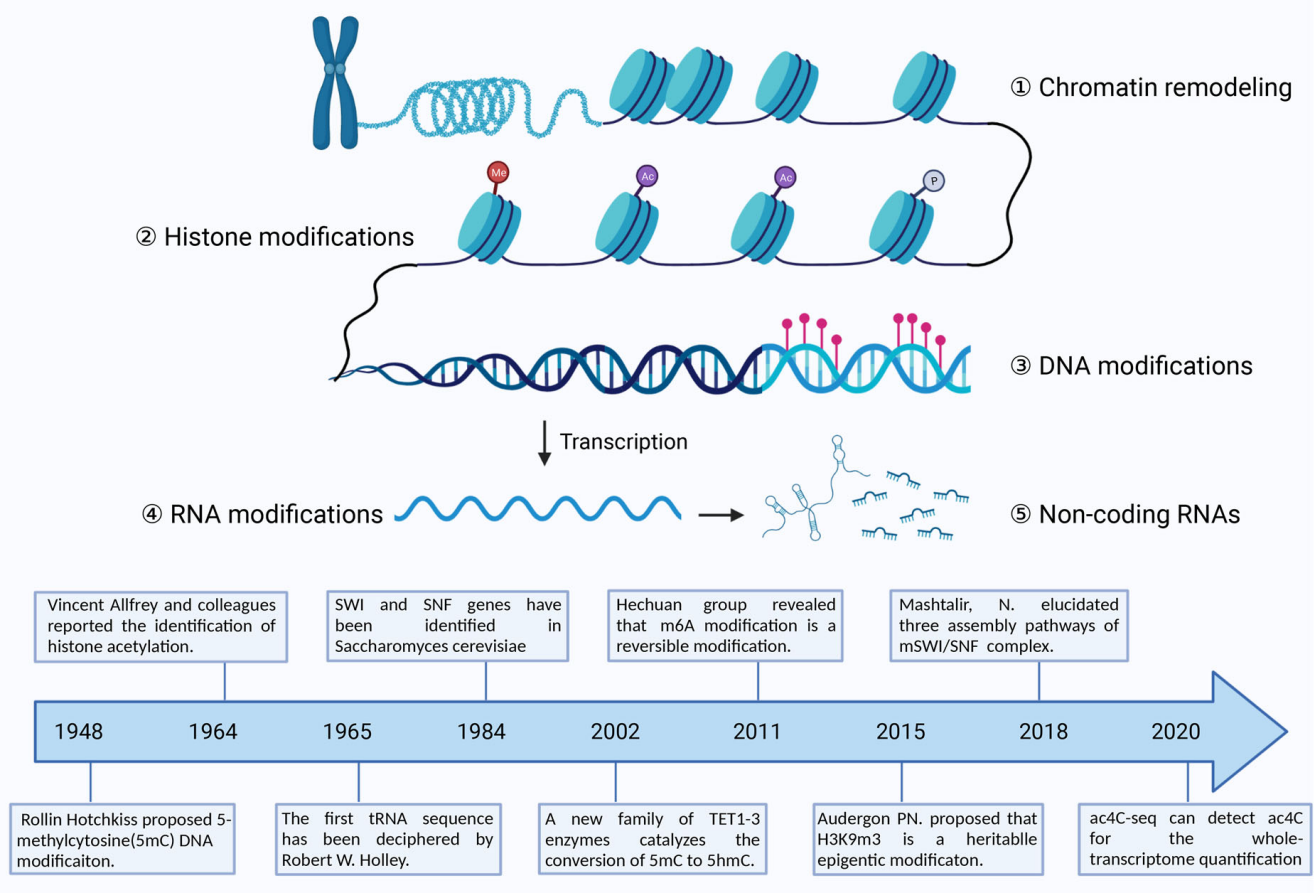
Schematic diagram of epigenetic mechanisms. Epigenetic modification is defined as heritable alterations in gene expression without changes the genetic DNA sequence. Epigenetic modifications can be grouped in five categories including chromatin remodeling, Histone modification, DNA modification, RNA modification, and noncoding RNA. The timelines show important research of each category. Abbreviation: ac4C, N4‐acetylcytidine; m6A, N6‐methyladenosine; *SNF*, sucrose non‐fermentable; *SWI*, switch; 5hmc, 5‐hydroxymethylcytosine (created with BioRender.com).

Different chromatin remodeling factors have similarities in protein structure and enzyme activity, and each has its own specificity. Based on functional analysis, ATP‐dependent chromatin remodeling factors can be roughly divided into four subfamilies: CHD (chromo domain helicase DNA binding), ISWI (imitation switch), SWI/SNF (switch/sucrose non‐fermentable), and INO80.^83^ SWI/SNF complex was discovered in *yeast* ^84^, ^85^ (Figure 3) and most studied in mammalian in recent years. Mashtalir et al.^86^ explained a new assembly pathway of mammalian SWI/SNF complex (mSWI/SNF) chromatin remodeling regulators (Figure 3), and concluded three distinct final assemblies: polybromo‐associated BAF complexes, BRGI/BRM‐associated factor complexes (BAFs), and a new defined non‐canonical BAFs. Gene mutations of mSWI/SNF subunits and histone modifications, variations, mutations on nucleosome can affect the activity of chromatin remodeling regulators and chromatin features,^87^ involving in the occurrence and development of many diseases. Exon sequencing revealed that mutations of gene encoding mSWI/SNF subunits occur nearly 25% in human cancer,^88^ such as typical SMARCB1 deficiency associates with numerous tumorigenesis. In addition, chromatin remodeling regulators also play an important role in regulating inflammatory responses. Upon stimulus, the mSWI/SNF complex cooperates with transcription factors to participate in the release of chemokines and cytokines from macrophages.^89^ Histone lysine‐specific demethylase 2b (KDM2B) interacts with Brahma‐related gene 1 (Brg1) subunit to mediate chromatin remodeling, promote IL‐6 production and inflammatory responses.^90^ Akirin2, a conserve nuclear protein, forms a complex by interacting with BAF60 proteins as well as IkB‐ζ, this complex links the SWI/SNF complex and NF‐κB to induce proinflammatory genes expression in macrophage including IL‐6, IL‐12b after LPS stimulus.^91^, ^92^

### 3.2 DNA methylation and inflammation

**FIGURE 4:**
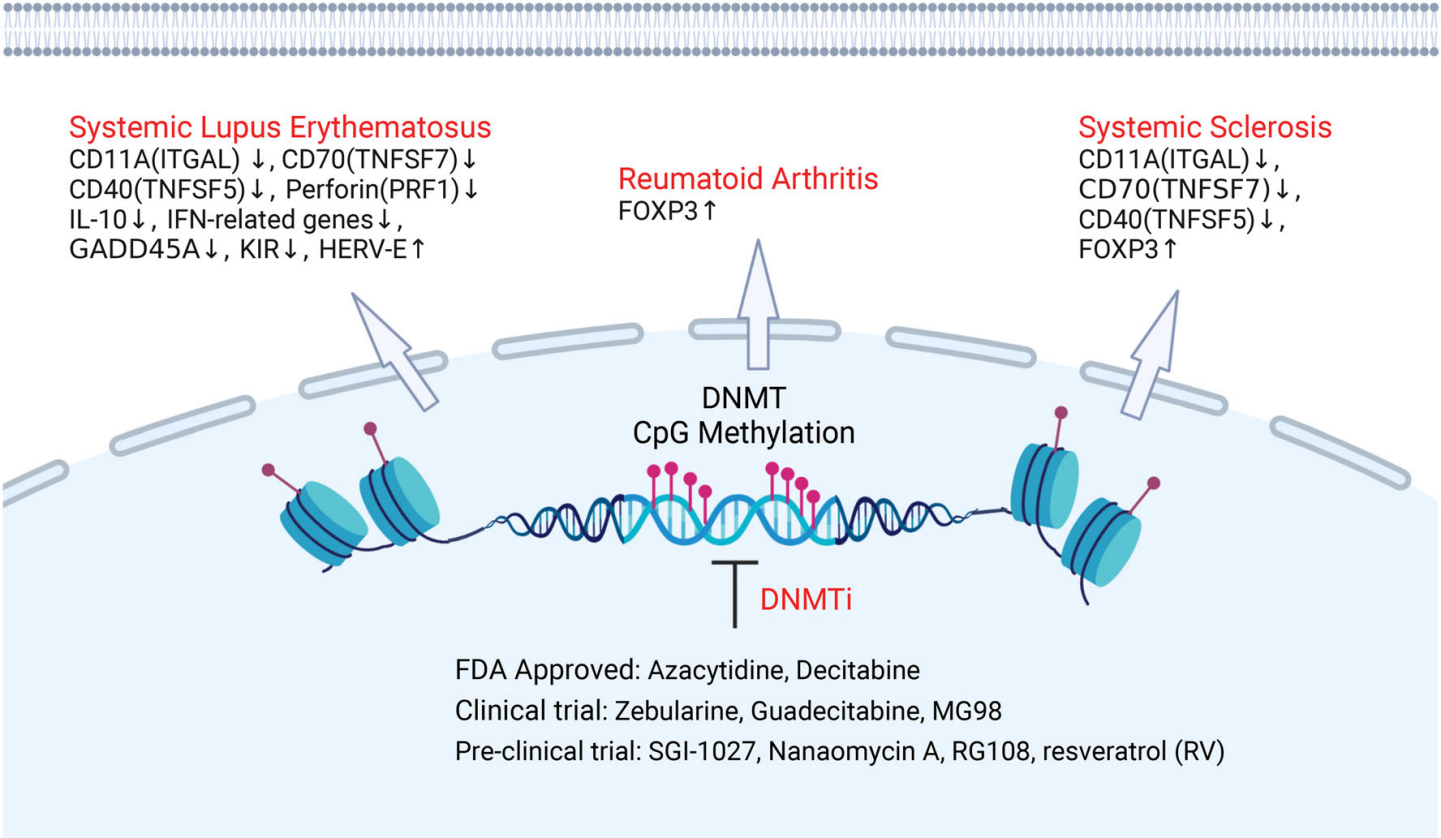
Schematic representation of DNA methylation involved in the pathogenesis of autoimmune diseases. Respectively, various hypomethylated genes in T lymphocytes of systemic lupus erythematosus patients were found, including *IL‐10*, *GADD45A*, interferon (IFN)‐related genes, *ITGAL*(*CD11A*), *PRF1* (perforin), *KIR*, *TNFSF7* (*CD70*), and *CD40LG* (*TNFSF5*). The downregulation of demethylating enzymes such as DNMT1, MBD3, and MBD4, leads to hypomethylation and overexpression of *TNFSF5*, *ITGAL*, and *TNFSF7* in T cells of systemic sclerosis. By contrast, the gene promoter of fork head box protein 3 (*FOXP3*) in CD4^+^ T cells of patients with systemic sclerosis and reumatoid arthritis is hypermethylated. Additionally, numerous molecules such as inhibitors to DNA methyltransferase (DNMT) have been screened for altering the DNA methylation status implicated in the inflammation disease (created with BioRender.com).

DNA methylation has precision regulatory functions in various biological processes, which can be stably inherited through meiosis, persists in mitosis, and is extremely important in maintaining genome integrity.^93^, ^94^ DNA methylation is catalyzed by DNA methyltransferases (DNMTs) and mostly found in CpG sites.^95^, ^96^ Mammalian genomes’ promoters show high CpG methylation levels, with approximately 70%–80% of CpG methylation.^94^ Hypermethylated CpGs are associated with genes that are silenced or have low expression.^97^ In general, DNA hypermethylation induces heterochromatin and impedes transcription factors binding with the promoter sites of numerous genes (Figure 4). It is supported that methyl‐CpG‐binding protein (MeCP‐1)‐deficient cells attenuated the repression of methylated genes, which was previously identified by Boyes and Bird.^98^ Indeed, DNA methylation played an important role in regulating inflammatory genes, as reviewed by Samanta et al.^21^ For instance, Shuto et al.^99^ observed that the expression of TLR2 in human cystic fibrosis bronchial epithelial cells was regulated by CpG methylation, which was correlated with a proinflammatory response to bacterial peptidoglycan. Another study indicated that downregulation of the TLR4 gene was mediated by both DNA methylation and histone deacetylation at the 5′ region of the TLR4 gene, which was required for maintaining homeostasis in the intestinal commensal system.^100^

5‐Methylcytosine (5mC) catalyzed by DNMT (Figure 3) was the most abundant type of DNA modification in the mammalian genome and proved by Hotchkiss.^96^ In mammals, DNMT family comprise three active members including two de novo DNA methylation enzymes, DNMT3A and DNMT3B, which contain a highly conserved DNMT domain (the MTase domain) in the carboxy terminus and two chromatin reading domains, ATRX‐DNMT3‐DNMT3L and Pro‐Trp‐Trp‐Pro motif (PWWP). DNMT1 is primarily a maintenance methyltransferase, methylating hemi‐methylated CpG dinucleotides in the nascent DNA.^94^, ^97^ In addition, DNMT3L, as an inactive DNMT, exerts important roles in regulation the activity of DNMT3A and DNMT3B in germline.^21^ DNA methylation by DNMTs (Figure 4) can also inhibit transcription through histone deacetylases (HDACs).^101^ Several lines of evidence indicated that Dnmt3a selectively upregulated the production of type I IFNs by maintaining a high expression of the HDAC HDAC9 rather than directly regulating the transcription of type I IFNs. Consequently, HDAC9 promoted the activation of TBK1 by maintaining its deacetylation status in response to innate stimuli.^102^, ^103^ Cooperatively, modifications in both DNA methylation and histone modifications also exert vital roles in regulating TNF‐α, which functions as a cytokine associated with inflammation.^104^, ^105^ A few examples of various studies that link DNA methylation to inflammation‐correlated diseases are outlined further below.

As an important epigenetic mechanism, DNA methylation is implicated in various biological processes of immune cells, including cell activation, proliferation, differentiation, and apoptosis, all of which are associated with the pathogenesis of inflammatory diseases.^106^ Recent studies have found that DNA hypermethylation at specific DNA sequences served as a biomarker for a broad variety of diseases.^107^ In AD, previous studies had demonstrated global methylation and hydroxy‐methylation alterations in the ADs human brain.^108^ Semick et al.^109^ identified that hypermethylation and hypomethylation were associated with brain region‐specific differences using microbead‐based methylome profiling of 73 postmortem samples. Similarly, Sanchez‐Mut et al.^110^ performed a comprehensive DNA methylation analysis of ND, including PDs, dementia with Lewy bodies, ADs, and Alzheimer‐like neurodegenerative profile associated with Down's syndrome samples. This finding suggested that similar pathogenetic mechanisms were associated with different neurodegenerative disorders.

As reviewed by Schmidl et al.,^111^ epigenetic mechanisms, also exerted important roles in controlling T‐cell responses. In RA (Figure 4), DNA methylation has been found to serve as a marker and regulates the relationship between genetic variants and patient outcomes.^112^ Another study involving changes in DNA methylation in RA indicated novel targets of DNA methylation‐ and miRNA‐associated dysregulation. Using a DNA methylation screening method, de la Rica et al.^113^ observed changes in novel key target genes, such as IL6R, DPP4, and CAPN8, as well as several HOX genes.

Demethylation in mammals is predominantly mediated by the TET methylcytosine dioxygenases family—namely TET1, TET2, and TET3.^93^ Tahiliani et al.^114^ revealed that the TET1 enzyme catalyzes the conversion of 5mC to 5‐hydroxymethylcytosine (5hmC) in 2009. Later discoveries had shown that TET proteins could oxidize 5mC to 5hmC, 5‐formylcytosine (5fC) and 5‐carboxylcytosine (5caC) by an iterative mechanism.^97^, ^115^ Multitudinous studies had indicated that aberrant regulation of DNA methylation was associated with the occurrence of many diseases.^93^, ^116^ Recent study had demonstrated that DNA methylation‐based biomarkers satisfied the criteria of a molecular biomarker of aging.^117^ In human cancer, DNA methylation crosstalked with H3K9 methylation to promote tumorgenesis.^94^ Aberrant DNA methylation was discovered in tumor‐infiltrating immune cell, DNMTi employed synergistically with immunotherapies by acting on both the cancer cells and immune cells to improve antitumor immune responses.^118^ DNA demethylation also plays a critical role in control of inflammation‐related gene transcription. For example, TET2 selectively mediates active repression of IL‐6 transcription during inflammation resolution in innate myeloid cells, including DCs and macrophages.^119^

Additionally, N6‐methyladenine (6mA), as a modification of distribution universally in the human genome, has drawn widespread attention since 6mA in various eukaryotic genomes was confirmed in 2015.^120^ A recent study suggested that N6AMT1 and ALKBH1 had been identified as methyltransferases and demethylases for 6mA modifications, respectively.^121^ However, the potential function and clinical significance of 6mA modification in human genomes still need further investigation.

### 3.3 Histone modifications and inflammation

The fundamental unit of chromatin is nucleosome, and the core region of nucleosome comprises histone octamers (two of each H3, H4, H2A, and H2B) that are enriched by abundant PTMs. Histone PTMs not only occur in the terminal tails, but on the lateral surface of nucleosome core regions.^122^ Precise and complex pathways involving enzymes that catalyze the formation of specific types of PTMs (writers) regulate the mechanisms of histone PTMs, and recognize particular PTMs via specific domains (readers) and remove PTMs (erasers). Histone PTMs exert important roles in the control of gene transcription, DNA replication, DNA repair, chromatin structure organization, and nucleosome dynamics. A variety of histone PTMs had been identified in recent years, mainly on lysine, including acetylation, methylation, ubiquitylation, and other long‐chain modification, such as crotonylation, benzoylation, and succinylation. Non‐lysine modifications include phosphorylation, serotonylation, s‐palmitoylation, and so on.^123^

#### 3.3.1 Histone methylation and inflammation

Due to its existence in three distinct states on both arginine (Rme1, Rme2 asymmetrical, and Rme2 symmetrical) and lysine (Kme1, Kme2, and Kme3) residues, methylation on histone tails represents a complex and more subtle chromatin modification than acetylation.^124^ This modification is tightly mediated by various methyltransferases and,, demethylases that act in concert to replace and remove specific methyl marks crucial for gene expression, genomic stability, and cell fate. Allis and coworkers identified the first histone methyltransferase that methylated specific residues in the histone tails, setting off a cascade of discoveries of histone demethylases.^125^, ^126^, ^127^

Lysine can be mono‐, di‐, or trimethylated by six major classes of histone lysine methyltransferase (KMT1‐6). KMT1 family that is in chargeof methylation of histone H3 at lysine 9 (H3K9) contains four members, including G9a, GLP,^128^ SUV39H1/2, and SETDB1.^129^ The methyltransferase KMT2 specifically performed the methylation of H3K4.^130^ KMT3 family comprises NSD1, NSD2, and NSD3, and primarily methylates H3K36. KMT4 only includes DOT1L.^131^ PRSet7 and SUV4‐20H1/2 are components of KMT5 family. EZH2 of KMT6 family is responsible for H3K27 mono‐, di‐, and trimethylation via its SET domain.^130^ Six family histone lysine demethylases have been identified in human cells that reverse lysine methylation since the discovery of the lysine demethylase LSD1. It has been shown that LSD1 (KDM1A) and LSD2 (KDM1B) from the KDM1 family demethylate H3K4me2/me1, and KDM2A and KDM2B from the KDM2 family methylate H3K36me2/me1 and H3K4me3 from the KDM2 family, respectively.^132^ The KDM3 family demethylases are classified into three subfamily members including KDM3A, KDM3B, and JMJD1C, with demethylase activities for H3K9me2/me1.^132^ KDM4A, KDM4B, KDM4C, and KDM4D are members of the KDM4 family, showing demethylase activities toward H3K9me3/me2 and H3K36me3/me2. The KDM5 family demethylases contain four subfamily members, namely KDM5A, KDM5B, KDM5C, and KDM5D, which are responsible for demethylation of H3K4me3/me2. The KDM6 family demethylases comprise UTY, UTX (KDM6A), and JMJD3 (KDM6B).^122^ The methylation of K79 of H3 has been the first studied modifications in the globular domain of histones. H3K79 methylation active gene expression in mammal at all their modification site.^133^

**FIGURE 5:**
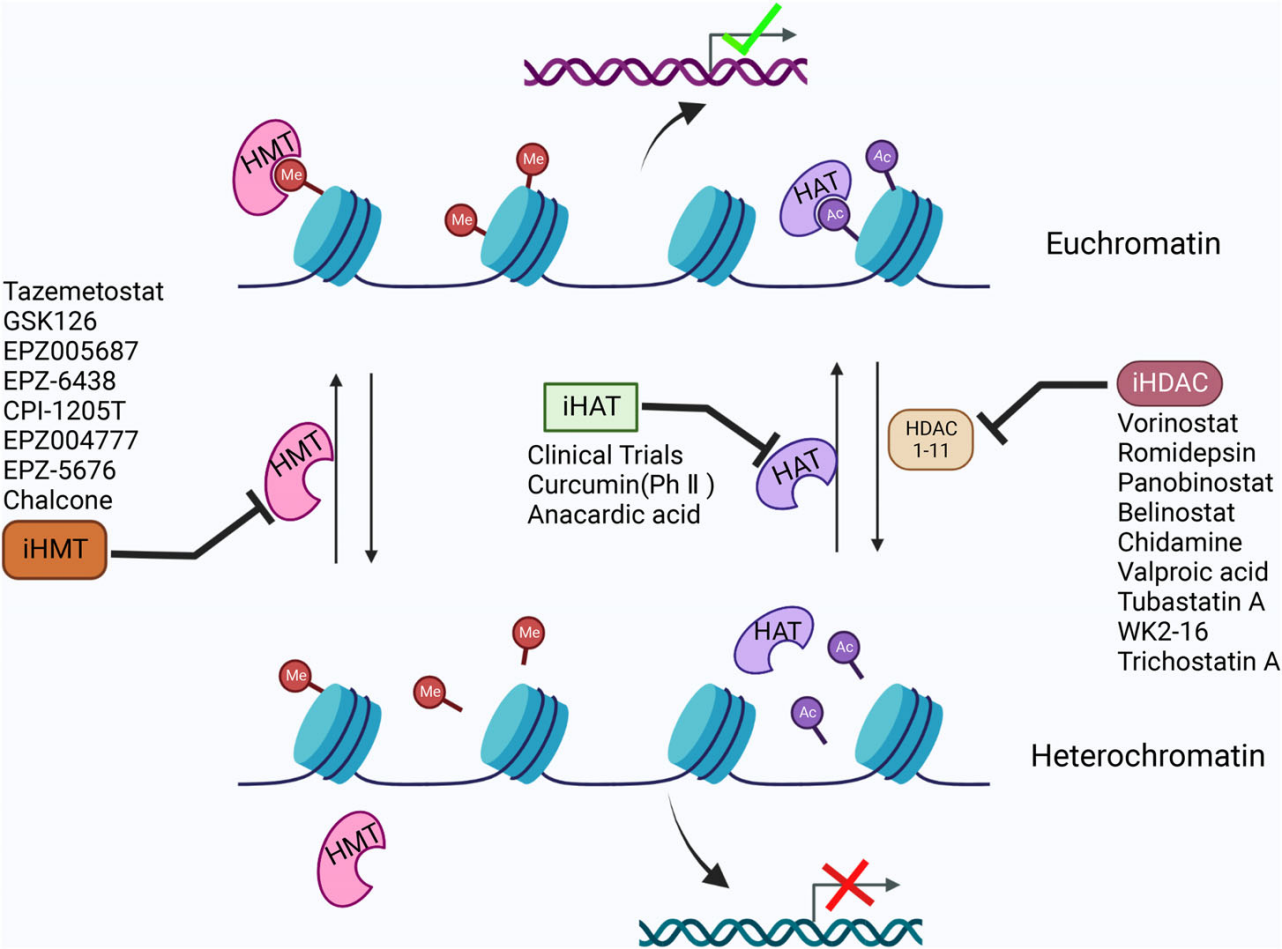
Inhibitors for targeting histone modifications regulators associated with inflammation response. Histone modifications mediated by histone acetyltransferases (HATs) (or HMT) and histone deacetylases (HDACs) result in activation chromatin state and repression chromatin state, respectively, which associated with inflammatory genes or genes responsible for innate immune response. Therefore, various small molecules and drugs are now under screening and trials to use them clinically to modify the altered epigenetic state and reduce the diseased condition (created with BioRender.com).

Histone methylation contributes to binding effector molecules with DNA in response to various cellular signals, which exert vital roles in gene transcription, repair, and replication processes.^134^ Similar to DNA methylation, it has been reported that histone methylation (Figure 5) was also involved in mediating inflammatory and anti‐inflammatory genes.^135^ For instance, Chauhan et al.^136^ observed the downregulation of the positive histone marks H3K4me3 and H3K9/14ac at the promoter region of proinflammatory genes, including *TNF‐α*, *IL‐6*, *NOS2*, *MHC‐II*, and a regulator of MHC‐II expression (*CIITA*). RelB a subunit of NF‐κB, directly binds and recruits G9a to IL‐1β promoter in THP‐1 endotoxin tolerant macrophages, forming a complex with HP‐1 and promoting gene silencing through H3K9 methylation.^135^ Additionally, H4K20me3 functions as a repression checkpoint and restricts the expression of TLR4 target genes in macrophages.^137^ Of interest, SET and MYND domain‐containing protein‐5 (SMYD5) methyltransferase was associated with NCoR corepressor complex maintaining H4K20me3 on TLR4‐responsive promoters, including *TNF‐α*, *IL‐1α*, *IL‐1β*, *CCL‐4*, and *CXCL10*.^137^

Foster et al.^138^ observed that transcription active mark, H3K4me3, was induced following LPS stimulation rather than at tolerizable gene promoters during endotoxin tolerance. In addition, H3K4me3 is involved in the regulation of LPS‐induced genes.^139^ Lysine‐specific methyltransferase Set7/9 (KMT7) functions as a novel coactivator of NF‐κB and positively modulates the expression of inflammatory genes via p65, which is associated with chromatin H3K4me in monocytes related to inflammation and diabetes.^140^ Similarly, Covic et al.^141^ found that the arginine methyltransferase CARM1/PRMT4 acted as a novel transcriptional coactivator of NF‐κB. PRMT4 cooperated with the transcriptional coactivator p300/CREB‐binding protein and the p160 family of steroid receptors as coactivators, resulting in the coactivation of NF‐κB‐mediated transactivation via H3R17 methylation in TNF‐α‐stimulated cells. In accordance, protein arginine methyltransferase 1 (PRMT1) synergistically coactivates NF‐κB‐dependent gene expression in concert with the transcriptional coactivators p300/CREB‐binding protein, PRMT4, and poly (ADP‐ribose) polymerase 1 at the macrophage inflammatory protein 2 and human immunodeficiency virus 1 long terminal repeat promoters in macrophages.^142^ Nevertheless, the nuclear chromatin mechanisms of modification regulated by PRMT1 are still unclear. Recently, Fan et al.^143^ identified that the protein arginine methyltransferase PRMT5 induced MHC‐II transactivation following IFN‐γ stimulation through histone H3 arginine 2‐dimethylated (H3R2Me2s) accumulation on the MHC‐II promoter along with CIITA in macrophages.

Recently, a plethora of research studies have drawn attention for the potential role of histone demethylases in catalyzing the removal of methyl groups from lysine and arginine residues in the regulation of inflammatory gene expression.^135^ The PHD Finger Protein 2 (PHF2) demethylase has been implicated in inflammatory responses in macrophages and contributes to the activation of TLR4‐responsive promoters through the removal of the H4K20me3 repressive mark.^137^ In addition, JMJD3, a Jumonji C family histone demethylase, exerts vital roles in controlling inflammation by reprogramming the epigenome, which is involved in the regulation of cell differentiation and cell identity in macrophages.^144^ De Santa et al.^144^ identified that JMJD3 contributed to the demethylation of the histone mark H3K27 but not H3K9. JMJD3 was first implicated in controlling inflammatory responses, binding polycomb group target genes and regulating their H3K27me3 levels and transcription. In a human monocytic cell line that performed gene networking and inflammatory pathway analysis, Das et al.^145^ found that JMJD3 depletion promoted the global levels of repressive histone H3K27me3 and downregulated several NF‐κB‐mediated inflammatory genes, including chemokines, cytokines, and immune receptors. JMJD3 has also been induced with post‐IL‐4 treatment in a STAT6‐dependent manner and subsequently accompanied by downregulation of H3K27 methylation on the promoters of M2‐regulating genes. In addition, STAT1 and STAT3 cooperated with JMJD3 to regulate the expression of proinflammatory genes following LPS treatment in rat microglial cells.^146^ Together, above study clearly suggest the dynamic roles of histone methylation during the inflammatory response.

#### 3.3.2 Histone acetylation and inflammation

Histone acetylation has a powerful function in regulating chromatin structure through neutralizing positively charged lysines and recruiting bromodomain‐containing proteins (BRDs) to promote the opening of chromatin, then allowing access to transcription factors and other transcription coactivators.^147^, ^148^ Allfrey et al.^149^ (Figure 3) reported the identification of histone acetylation and correlated this protein modification with transcriptional regulation. Acetylation of histone H3 or H4 tails is reversibly controlled by two groups of enzymes: histone acetyltransferases (HATs) and HDACs.^150^ Based on their primary structure homology, HATs have been identified and grouped into three major families, including GNAT, MYST, and p300/CBP.^151^ And HDACs are divided into four major families, including Class I (HDACs 1, 2, 3, and 8), Class II (HDACs 4, 5, 6, 7, 9, and 10), Class III or sirtuins (SIRT1‐7), and Class IV (HDAC11).^122^ Acetylation of histones, a more dynamic process, is closely coupled with HATs and histone deacetyl transferases (Figure 5), which have both activating and repressing roles^152^ and play complex roles in inflammation induced by different stimuli, such as chemokines, LPS, IL‐1β, and TNF‐α.^153^ Many of these stimuli exert vital roles via the induction of proinflammatory transcription factors, such as AP‐1 and NF‐κB, and other intracellular kinase pathways, such as MAPKs and PI3K.^154^ In this context, Sullivan et al.^104^ stated that LPS stimulation increased the H3 and H4 acetylation of the TNF‐α locus, resulting in induction of gene transcription. Xing et al.^155^ observed that the activation of HDACs induced by LPS contributed to Thy‐1 gene expression and lung fibroblast proliferation via TLR4 signaling. Increased IFN‐β production has been observed via ASF1a‐mediated H3K56ac modification in vesicular stomatitis virus‐infected macrophages.^156^ In addition, LPS stimulation is involved in the regulation of proinflammatory gene expression in macrophages but affects the expression of almost all HDACs to different magnitudes and kinetics.^157^ For instance, HDAC1 is gradually upregulated to 8 h after LPS stimulation with stabilization after 24 h. In contrast, HDAC6, 10, and 11 are gradually downregulated until 8 h and recover slightly by 24 h after stimulation. Of interest, HDAC4, 5, and 7 display the same expression pattern but are upregulated 24 h after LPS stimulation.^158^

HDACs have been implicated in the regulation of proinflammatory genes, and they reverse the activity of HATs by removing acetyl groups from histones, resulting in the condensation of chromatin and the repression of inflammatory gene transcription (Figure 5). For instance, Villagra et al.^158^ reported that CBP/p300 could rapidly acetylated the promoters of several proinflammatory cytokines, such as IL‐1, IL‐2, IL‐8, and IL‐12, resulting in transcriptional activation and displaying decreased HDAC activity. The activation of NF‐κB signaling mediated by HDACs plays a central role in mediating inflammatory responses. Zhong et al.^159^ stated that binding HDAC1 with the p50 subunit of NF‐κB repressed NF‐κB‐dependent gene expression in unstimulated cells. However, after activation, NF‐κB complex containing phosphorylated p65 associated with CBP translocate to the nucleus and displaced p50/HDAC1 complex. A similar mechanism not restricted to HDAC1 as HDAC3 has been demonstrated to interact with p50.^160^ Upon IL‐1 stimulation, the corepressor complex associated with HDAC3 in concert with TAB2 and N‐CoR translocates from the nucleus to the cytoplasm through MEKK1.^160^ Nonetheless, a physical interaction between TAB2 and N‐CoR for HDAC1 complexes has not been reported. In addition, the lysine 221 of RelA (p65) was also deacetylated by HDAC3 in the nucleus, which promoted NF‐κB binding to IκBα and its nuclear export.^161^ Thus, the use of these enzymes in the regulation of pro‐ and anti‐inflammatory mediators in response to LPS stimulation has promoted the use of selective HDAC inhibitors (Figure 5) for mediating the immune response.^158^

#### 3.3.3 Histone ubiquitination and inflammation

Histone ubiquitylation, like other modifications, also plays an important role in regulating gene transcription and chromatin structure in mammals.^122^ The difference is that cascade reactions perform these PTMs through a series of ubiquitin enzymes, including E1 activate, E2 conjugate, and E3 ligase enzymes. Ubiquitin enzymes conjugate ubiquitin (Ub) on a lysine residue or on Ub itself to form mono‐ubiquitination or poly‐ubiquitination of histone proteins. Mono‐ubiquitination of H2A and H2B are the most abundant mono‐ubiquitination of histone, leading to H2AK118/119ub1 and H2BK120ub1, respectively.^162^ H2BK119ub1 is implemented by RING1A/B in the PRC1 complex,^163^ and is essential for polycomb group proteins to maintain transcriptional repression. Tamburri et al.^164^ reported that H2A K119ub1 deficiency induced a rapid displacement of PRC2 activity and a loss of H3K27me3 deposition. In addition, H2BK120ub1 is carried out by the UBE2A/B (RAD6) E2 ubiquitin conjugating enzyme and the RNF20/40 E3 ligase,^162^ H2B K120ub is involved into transcription elongation, which is also promoted by genotoxic agents and required for DNA double‐strand break (DSB) repair.^165^

Histone ubiquitination is also a reversible epigenetic modification, deubiquitinating enzymes are grouped into six subfamily members in human genomes: ubiquitin C‐terminal hydrolases, MCP 1‐induced protein, Josephins, JAB1/MPN/MOV34 family (JAMMs), ubiquitin‐specific proteases (USPs), and ovarian tumor proteases.^166^ Deubiquitinase Trabid promotes TLR‐induced histone modifications at the IL‐12 and IL‐23 promoters, which is involved into deubiquitination and stabilization of the histone demethylase Jmjd2d, impairing the differentiation of inflammatory T cells and protecting mice from autoimmune inflammation.^21^ USP39 significantly facilitates JAK/STAT downstream of type I signaling by enhancing IFN‐stimulated response elements’ promoter activity and expression of IFN‐stimulated genes.^166^

Mono‐ubiquitination exerts a critical role in transcriptional regulation, protein translocation, and DNA damage signaling. Several studies have revealed that mono‐ubiquitination of histone 2A (H2Aub) and histone 2B (H2Bub) were widely involved in the development of inflammation progression. Whereas, H2Aub is typically correlated with gene silencing, and the latter is more often associated with transcription activation.^167^ For example, E3 ligase RNF20 restricts NF‐κB target gene transcription upon TNF‐α treatment in non‐transformed human mammary epithelial MCF10A cells. In mice and humans, downregulation of RNF20 and H2B mono‐ubiquitination result in enhancing chronic colonic inflammation and inflammation‐associated colorectal cancer, partly by augmenting NF‐κB activity and attenuating the antitumoral T‐cell response.^168^ Furthermore, recent study has revealed that a natural small‐molecule epoxymicheliolide (ECL) directly bound to histone H2B to inhibit MG‐mediated neuroinflammation via recruiting E3 ligase RNF20.^169^

The mono‐ubiquitination of histone H2B at lysine 120 (H2B K120ub1) mediated by the ubiquitin ligases RNF20 and RNF40 was shown to play context‐dependent roles in the development of inflammation. Koskinsky et al.^170^ reported that loss of H2B mono‐ubiquitination facilitated intestinal inflammation through reducing vitamin D receptor activity, identifying RNF20 and RNF40 as critical mediators of inflammatory bowel disease. Of note, Pandey et al.^171^ disclosed that H2A lysine 119 mono‐ubiquitination (H2A K119Ub) partially regulated macrophage infiltration mediated by angiotensin II in type 2 diabetic nephropathy (T2DN) through both AT1 and AT2 receptors. Furthermore, there is also crosstalk between ubiquitination and other histone modifications such as histone acetylation during inflammation progression. For examples, Wei et al.^172^ demonstrated that the ubiquitylation and deubiquitylation of CREB‐binding protein regulated by FBXL19 and USP14, respectively, result in the modulation of histone acetylation such as H4K12ac and H4K8ac, the expression of cytokine‐encoding genes, and lung inflammation. In addition, USP38, a novel histone deubiquitinase, has been reported working together with the histone H3K4 modifier KDM5B to orchestrate inflammatory responses.^173^ Specifically, H2B K120ub1 removed by USP38 resulted in recruitment of demethylase KDM5B to the promoters of proinflammatory cytokines IL‐6 and IL‐23a with LPS treatment. In turn, KDM5B reduced H3K4me3 leading to inhibition of the binding of NF‐κB transcription factors to the IL‐6 and IL‐23a promoters.^173^ Thus, these results suggest that mono‐ubiquitination of histone H2A and H2B could act as a potential pharmacological target to develop small‐molecule drugs against MG‐mediated neuroinflammation.

#### 3.3.4 Histone phosphorylation and inflammation

Histone phosphorylation take place on serine, threonine, and tyrosine residues,^21^ the tails of histones become negatively charged, thus altering chromatin structure and transcription factors interactions.^122^ Histone phosphorylation is involved in many cellular processes, such as DNA repair, chromosome condensation, gene transcription, and cell apoptosis. Different kinases phosphorylate histone tails, and the phosphate groups are removed by phosphatases. There are two major classes of phosphatases, including the Ser/Thr phosphatases and tyrosine phosphatases (PTPs). PTPs are composed of three separate subfamilies, the cysteine‐based PTPs, aspartic acid‐based PTPs, and histidine‐based PTPs.^174^ Of these, dual‐specificity phosphatases are type I cysteine‐based PTPs that have been extensively studied in human cancer.^175^ H2AX at serine 139 (γH2AX) is the first histone PTM to show specific induction at DNA DSBs, which can be distributed over large (up to 2 Mb) domains that form foci for the DNA damage response.^123^ Various kinases can mediate the phosphorylation on this special site including ataxia‐telangiectasia mutated (ATM), ATM and RAD3‐related, and DNA‐dependent protein kinase catalytic subunit.^122^ Orlando et al.^176^ demonstrated that phosphorylation of γH2AX inhibited the differentiation process of human stem cells into progenitor cells, and reducing the phosphorylation level of γH2AX promoted the progress, providing a mechanism to target self‐renewal. During mitosis, histone phosphorylation is critical for protein recruitment and regulation, the spindle assembly checkpoint kinase Bub1 mediates histone H2A Thr‐120 phosphorylation and subsequent recruitment of Sgo1 to the centromeric region.^177^ Furthermore, histone H3.3 is an ancestral variant of histone H3, and enriched at enhancers and active genes. H3.3 Ser‐31 phosphorylation stimulates activity of the acetyltransferase p300 in *trans*, and promotes acetylation on histone H3 at lysine 27 (H3K27ac) at mESC enhancers, suggesting that H3.3 plays an important role in guiding gene transcription.^178^ Armache et al.^179^ found that phosphorylation of Ser‐31 of H3.3 allowed transcription apparatus to access stimulation‐induced genes with greater efficiency. Following LPS stimulation, histone H3 phospho(Ser10)‐acetylation(Lys14) (H3S10phK14ac) was increased in the hypothalamus and hippocampus, and enriched at the promoters of IL‐6, c‐Fos, and iNOS genes, inducing neuroinflammatory response of neurons and reactive MG.^178^ In the TAM, Banerjee et al.^180^ deciphered an unexplored TLR signaling that ERK‐1/2 activation in a MyD88‐independent pathway lead to transcription favorable histone modification at the IL‐10 promoter region to enhance IL‐10‐mediated immunosuppression.

### 3.4 RNA modifications and inflammation

All known RNA species can be the targets of modifications, with ribosomal RNA (rRNA) and transfer RNA (tRNA) being the most modified.^181^ Over 100 types of RNA modifications have been identified, and it was generally several finely regulated molecular processes including RNA metabolism, decay, splicing or translation, localization, stability, turnover, and binding to RNA‐binding proteins (RBPs) or other RNases. N6‐methyladenosine (m6A), mainly a posttranscriptional epigenetic modification of RNA, mediates its effect on many steps of RNA metabolism, including mRNA translation, mRNA decay, mRNA export from the nucleus to the cytoplasm, mRNA degradation, and the biogenesis of miRNAs and lncRNAs.^182^, ^183^, ^184^ m6A RNA methylation (Figure 3) mediates its effect through three groups of enzymes, that is, writers, erasers, and readers. Recent studies have shown the impact of m6A RNA modification on various inflammatory states, including metabolic disease, ND, autoimmunity, infection, and cancer.^185^ For example, METTL3, the core methyltransferase of m6A, has been found to inhibit the inflammatory response induced with LPS by exerting anti‐malabsorption of long‐chain fatty acid activity in vitro.^186^ Zong et al.^186^ observed that METTL3 depletion suppressed the expression of TRAF6 by downregulating the m6A level of TNF receptor associated factor 6 (TRAF6) mRNA, resulting in the suppression of the NF‐κB and MAPK signaling pathways.

Recently, it was found that the levels of m6A and METTL3 expression were upregulated in human dental pulp cells (HDPCs) upon LPS stimulation. Similarly, deletion of METTL3 in HDPCs following LPS treatment increases the expression of MyD88S, leading to a decrease in the expression of inflammatory cytokines and suppressing the activation of the NF‐κB and MAPK signaling pathways.^187^ In contrast, depletion of YTHDF2 promotes the expression of MAP2K4 and MAP4K4 mRNA by stabilizing mRNA transcription in LPS‐treated RAW 264.7 cells, thereby activating MAPK and NF‐κB signaling pathways, which further induces the expression of proinflammatory cytokines and aggravates the inflammatory response.^188^ It has been reported that m6A modifications were linked to proinflammatory genes in the TME. For instance, the levels of m6A modification and mRNA expression are increased in hepatocellular carcinoma (HCC). LPS stimulation increases the m6A methylation of G‐protein alpha‐subunit (GNAS) mRNA in HCC cells, increasing GNAS expression, and its high expression subsequently promotes the growth and invasion of HCC cells by interacting with STAT3.^189^ In addition, numerous studies have suggested that m6A modification of noncoding RNAs (ncRNAs) could affect tumor formation. METTL3 may have an oncogenic role in bladder cancer by interacting with the microprocessor protein DGCR8 and positively modulating the pri‐miR221/222 process in a m6A‐dependent manner.^190^

Similar to modifications that occur on DNA, RNA can be methylated at position 5 of cytidine residues. RNA 5mC has been identified and found on mRNA, rRNA, ncRNA, tRNA, and enhancer RNA (eRNA). The enzymes are responsible for 5mC modification of RNAs including seven members of the NOL1/NOP2/SUN domain (NSUN) family, namely NSUN1 to NSUN7, and DNMT‐like 2 (DNMT2). 5mC in RNA can be oxidized by Tet‐family enzymes to 5hmC.^191^ Other RNA modifications have been reviewed in detail, including h5mC, ac4C, m7G, pseudouridine, and uridine.^181^ N1‐methyladenosine (m1A) has been mainly characterized in tRNAs and was also a dynamic and reversible modification. The methyltransferases catalyzing m1A on tRNA are tRNA methyltransferase 10 homolog A (TRMT10A) and the tRNA methyltransferase noncatalytic subunit 6 (TRM6)—tRNA methyltransferase catalytic subunit 61 (TRM61) complex, and the latter also acts on mRNA.^181^ ALKBH1, ALKBH3, and FTO can erase m1A from RNA.^95^ m1A maps uniquely to positions near the translation start site and first splice site in coding transcripts and correlates with upregulation of translation in general.^192^

### 3.5 Noncoding RNA, epigenetics, and inflammation

Based on the Encyclopedia of DNA Elements (ENCODE) project, ∼80% of the human genome is transcribed into various ncRNAs,^193^ some of which have been shown to play key roles in both normal cellular function and diseases and have emerged as a major source of biomarkers targeting clinical therapies.^194^ Thus, the regulatory role of ncRNAs in inflammation‐related diseases cannot be ignored. The first ncRNA—tRNA has been identified by Robert W. Holley and ncRNAs (Figure 3) are classified into several types based on size, including lncRNAs, circular RNAs (circRNAs), miRNAs, and other types, such as piwi‐interacting RNAs (piRNAs), small interfering RNAs, small nuclear RNAs, and small nucleolar RNAs (snoRNAs).^195^

#### 3.5.1 miRNA and inflammation

miRNAs, a novel class of endogenous ncRNAs of 18–25 nucleotides, are involved in the regulation of messenger RNA stability and translation at the posttranscriptional level in eukaryotic organisms.^196^, ^197^ miRNAs have been the most widely studied ncRNA over 20 years, since the first miRNA (lin‐4) was identified in *Caenorhabditis elegans* in 1993 and the first mammal miRNA (let‐7) was discovered in 2000.^198^, ^199^ Most miRNAs are encoded by intronic regions of genes and begin with transcription by RNA polymerase II (Pol II). The maturation processes of miRNAs are regulated by four key enzymes: Drosha, exportin 5, Dicer, and Argonaute 2 (AGO2).^200^, ^201^ Functional studies have revealed that miRNAs played a central role in cell proliferation, differentiation, apoptosis, and development, and dysregulation of miRNAs functions lead to human diseases, including cancer,^202^ ND,^203^ and SLE.^204^

**FIGURE 6:**
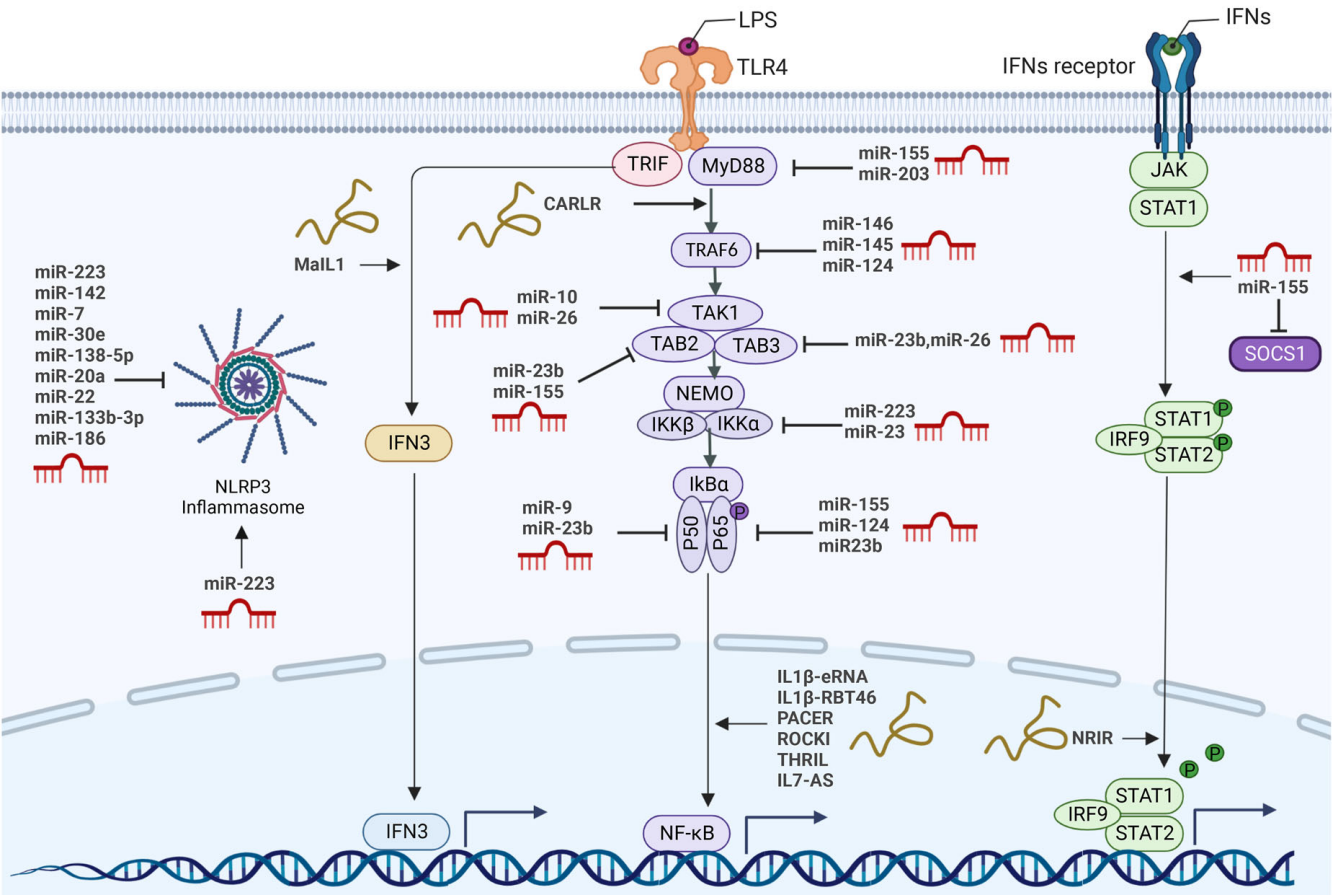
Regulation of pattern recognition receptors‐triggered phagocyte immunity by immuno‐noncoding RNA. Noncoding RNA has been implicated in NLRP3 (Nod‐like receptor family pyrin domain containing 3) inflammasome, PRR‐TRIF‐IRF3, PRR‐MyD88‐NFκB, and JAK/STAT‐dependent interferon‐stimulated genes (ISGs) expression induced upon interferons (IFNs) stimulation. In human phagocytes, cytoplasmic and nuclear long noncoding RNAs (lncRNAs) are recognized to be negative and positive regulators of signaling pathways involved in innate immune responses. Of note, modulatory effects of microRNAs (miRNAs) on acting as positive and negative regulators of these pathways in immune cells are indicated (created with BioRender.com).

Baltimore's group first reported that PAMP recognition, such as microbial cell wall components or viral nucleic acids or bacteria, was coupled with the expression changes of various miRNAs.^205^ Ever since, it has been the subject of countless research projects that investigated the roles of miRNAs in inflammatory and autoimmune diseases (reviewed in Refs.^197^, ^206^, ^207^). The inflammatory response harmonizes the activation of various signals, modulating the expression of both anti‐ and proinflammatory regulators. The nuclear factor NF‐κB pathway (Figure 6), as a prototypical proinflammatory signaling pathway, has exerted complex roles in inflammation, largely based on NF‐κB roles inducing the expression of proinflammatory genes, including cytokines, chemokines, and adhesion molecules.^28^

Upon stimulation of cell surface receptors, such as TLRs, tumor necrosis factor receptors, IL‐1 receptors and receptor for advanced glycation endproducts, miR‐146a and miR‐146b, which are located on chromosomes 5 and 10 in human, respectively, are both upregulated.^208^, ^209^, ^210^, ^211^, ^212^ Taganov and coworkers have identified that the human monocytic cell line THP‐1 stimulated with LPS from *Escherichia coli* increased the expression of miR‐146a/b, miR‐155, and miR‐132.^208^ miR‐146a is induced by various microbial components and proinflammatory mediators (e.g., LPS, IL‐1β, and flagellin) and functions as an immediate early response gene. The elevation of miR‐146a expression mediated by LPS was identified to depend on NF‐κB‐binding sites in its promoter region. Their research suggested that interleukin 1 receptor associated kinase 1 (IRAK1) and TRAF6 represent potential molecular targets of miR‐146 posttranslational repression, which is a novel mechanism of negative feedback regulation of TLR‐mediated NF‐κB activation.^208^ miRNA‐146a has also been upregulated during viral infection and act as a feedback negative regulator of the RIG‐I‐dependent antiviral pathway by targeting TRAF6, IRAK1, and IRAK2.^213^

miR‐155, similar to the function of miR‐146 in TLR‐induced inflammation, was also considered a feedback negative regulator of the TLR‐NF‐κB axis signaling pathway in human and murine macrophages and monocytes (Figure 6). Various searches have revealed that miR‐155 acts as a negative regulator and targets several key molecules, including IKKe,^213^ C/EBPb^214^ and p65,^215^ TAB2,^216^ and MyD88,^217^ which are involved in the TLR signaling pathway (reviewed in more detail in Ref.^218^). Moreover, miR‐155 affects proinflammatory signaling pathways, exerts negative roles in the regulation of TNF signaling and targets the signaling components TAB2, IKKε, and NIK.^219^ Nevertheless, miR‐146 only targets the TLR4 signal transduction components TRAF6 and IRAK1 (Figure 6) upon LPS stimulation, despite the general activation of two miRNAs through the NF‐κB pathway.^219^ Various studies have demonstrated that miR‐146 and miR‐155 were involved in T‐cell immunity.^220^, ^221^, ^222^ Interestingly, miR‐146 was shown to inhibit IFN‐γ production by T cells,^223^ while miR‐155 was found to counter‐regulate IFN‐γ production by Th1 cells.^224^ Moreover, targeting SOCS1 by miR‐155 (Figure 6) enhanced the inflammatory responses of macrophages and type I IFN signaling in biliary atresia,^225^ which suggested that miR‐155 exert proinflammatory functions. Together, the above‐delineated studies suggest that both miR‐146 and miR‐155 exert complex roles in inflammation and have at least partly opposing functions in T‐cell immunity.

In a follow‐up study, further miRNAs were shown to have well‐documented functions in TLR‐induced inflammatory responses (Figure 6). Besides miR‐146 and miR‐155, miRNA‐223 also functions as a classical TLR‐responsive miRNA, which target IKKα (Figure 6) to attenuate the TLR9/NF‐κB signaling pathway in neutrophils.^226^, ^227^ Neudecker et al.^228^ described an intercellular transfer of miR‐223 from polymorphonuclear neutrophils to epithelial cells, resulting in attenuating lung inflammation through repression of PARP‐1. It is also demonstrated that STIM1 mediated NLRP3 expression and inflammasome activation by targeting miR‐223 in IAV‐induced inflammatory injury of lung epithelial cells.^229^ Further study demonstrated that miR‐223 regulated inflammation and the NLRP3 inflammasome by targeting various factors, including TRAF6, IKKα, TLR4, CXCL2, CCL3, IL‐6, and IFN‐I.^230^ These studies suggest that miR‐223 may be considered as a diagnostic biomarker and therapeutic target in COVID‐19 or others.

miR‐203, the first skin‐specific miRNA, is involved in regulating various physiological and pathological processes (Figure 6). It has been indicated that miR‐203 controlled several key signaling pathways coupled with innate immune activation. miR‐203 negatively regulates the expression of the nuclear factor interleukin‐3 (NFIL3) by binding with NFIL3 3′‐untranslated regions (3′‐UTR), which speeds up apoptosis and inflammation induced by LPS in cardiomyocytes.^231^ Moreover, miR‐203 is highly induced in mouse lung tissue upon LPS stimulation^232^ and negatively regulates ischemia‐induced microglial activation by targeting the TLR adaptor protein MyD88.^233^ Additionally, our previous studies have shown that miR‐203 targeted insulin receptor substrate 1 and inhibited cell proliferation and the ERK pathway in prostate cancer.^234^ Focusing on the role of miR‐203 in fine‐tuning the innate immune response in skin, Primo and coworkers confirmed that miR‐203 could directly target IL‐24, TNF‐α, and SOCS3.^235^ Thus, miR‐203 could be regarded as a key anti‐inflammatory miRNA. Together, targeting specific miRNAs might be a novel therapeutic strategy based on the roles of anti‐ and pro‐inflammation in human innate immunity. Many further roles of miRNAs in immune cell function have been reviewed.^197^, ^236^, ^237^ Together, therapeutic strategies to target specific miRNAs in vivo might open novel treatment options in microbial and viral infection.

#### 3.5.2 Long noncoding RNA and inflammation

LncRNAs, which are broadly distributed in mammals, are the largest portion of the noncoding transcriptome with little or no protein translation capacity. LncRNAs are over 200 nucleotides and have been proposed to participate in a variety of biological processes through complicated mechanisms.^238^ To better understand lncRNA functions, Kopp and Mendell^239^ classified these transcripts into two groups depending on their subcellular location: those that regulate gene expression and/or local chromatin remodeling in *cis* virus and those that leave the site of transcription and carry out their functions throughout the cell in *trans*. Interestingly, the interactions of lncRNAs with mRNAs can significantly affect mRNA stability, translation or isolation. Additionally, the interaction between lncRNAs and multiple proteins can strengthen the assembly of protein complex or weaken the interaction between proteins. Recent studies have even revealed that lncRNAs could act as precursors of miRNAs or circRNAs.^195^

Similar with miRNAs, accumulating evidence has indicated that lncRNAs were involved in both inflammatory pathways and immune reactions. Unlike the intensively studied miRNAs, most lncRNAs seem to control various cellular processes ranging from the cytosolic receptor signaling cascade to the opening or closing of local chromatin structures in *cis* and in *trans* by establishing agonistic and antagonistic interactions with proteins.^240^ Several lines of evidence suggest that lncRNAs have been implicated in negative and positive regulation of innate immune gene expression in response to inflammatory signals, including TLR ligands and cytokines (Figure 6). We will expand on this.

Within the IL‐1β locus, for instance, the lncRNAs IL‐1β‐eRNA and IL‐1β‐RBT46 (Figure 6) promote messenger RNA transcription induced by bacterial LPS and the expression of the proinflammatory cytokine and IL‐1β through the TLR4‐MyD88‐NF‐κB axis in human monocytes.^241^ Krawczyk and Emerson^242^ described that the nuclear lncRNA PACER (p50‐associated Cox‐2 extragenic RNA) was expressed upon induction of LPS and bind with p50, a repressive subunit of NF‐κB, resulting in suppression of p50 homodimer formation and facilitating NF‐κB p50/p65 heterodimer formation. Similar to PACER, lncRNA CARLR has been shown to act as a potential novel player in the NF‐κB inflammatory pathway (Figure 6). Upon LPS stimulation, the lncRNA CARLR is inducible in human macrophages and facilitates proinflammatory gene expression by binding with the NF‐κB member p65.^243^ Follow‐up studies indicated that both lncRNA ROCKi and IL‐7‐AS (Figure 6) were implicated in the regulation of TLR‐MyD88‐dependent gene expression in human macrophages.^244^, ^245^ In contrast to lncRNAs that follow TLR4‐MyD88‐NF‐κB activation, IL‐7‐AS, which associates with p300 and SWI/SNF, promote histone acetylation and promoter remodeling to facilitate inflammatory gene transcription.^244^

Another signaling pathway in response to LPS is the TOII/IL‐IR domain‐containing adaptor‐inducing IFN‐β (TRIF)‐dependent pathway (Figure 6), which mediates the expression of IFNs and stabilizes the transcriptional activity of NF‐κB.^246^ It has shown that TLR4 needed to be delivered to specific region after ligand binding to facilitate signal transduction in both the MyD88‐ and TRIF‐dependent pathways (Figure 6).^246^ Recent evidence has revealed that several lncRNAs were involved in IFN production and the induction of downstream ISGs in the innate immune system. The lncRNA MaIL1 is a critical structural component of the TLR4‐TRIF pathway (Figure 6), which activates type I IFN production, and it can form a complex by binding with the ubiquitin‐adapter optineurin (OPTN) in the cytoplasm, which is essential for OPTN‐TBK1 kinase‐dependent IRF3 transcription factor phosphorylation.^247^ Similarly, upon coactivation with LPS and IFN‐γ, the lncRNA negative regulator of the IFN response (NRIR) was shown to be significantly upregulated in human macrophages (Figure 6).^247^ Mariotti et al.^248^ revealed NRIR was upregulated in monocytes from systemic sclerosis patients, which was positively associated with the IFN score of systemic sclerosis patients. Hence, within TLR4‐activated primary human phagocytes, both lncRNAs MaIL1 and NRIR function to leverage the IFN‐associated immune response. Expanding reports have indicated that lncRNAs contributed to regulating neuroinflammatory pathways in the CNS, including neurodegeneration, infection, stroke, and neuropathic pain.^249^ The lncRNA neuroblastoma differentiation marker 29 (NDM29) transcribed by RNA pol III is highly induced in the cerebral cortices of AD patients.^250^ Upon stimulation with IL‐1α, TNF‐α, and LPS, upregulation of NDM29 was associated with an increase in the formation of total β‐amyloid and amyloid precursor protein synthesis.^250^ Recent reports have indicated that both miR‐155‐5p and lncRNA CTB‐89H12.4 function as new potential therapeutic targets of ADs for using bioinformatics approaches, and the overexpression of miR‐155‐5p leads to downregulation of the expression of lncRNA CTB‐89H12.4, which competitively binds to miR‐155‐5p.^251^

Inflammatory stimulation, for example, with diverse reagents, such as LPS and IFN‐γ, results in the induction of lincRNA‐p21 via the p53‐dependent pathway in microglial cells.^252^ LincRNA‐p21 functions as a ceRNA targeting protein kinase C (PKC‐δ) mRNA and binds to the miR‐181 family, resulting in the promotion of microglial activation‐induced neurotoxicity.^252^ Similar to lincRNA‐p21, the lncRNA snoRNA host gene (SNHG1) is induced by LPS and promotes the expression of IL‐6, TNF‐α, and IL‐1β through the miR‐7/NLRP3 inflammasome pathway in microglial cells.^253^ Wang and Zhou^254^ observed that lncRNA MALAT1 promoted H3 histone acetylation at the MyD88 promoter to facilitate the inflammatory response in MG by provoking MyD88/IRAK1/TRAF6 signaling. The more detailed roles of other lncRNAs in neuroinflammation are reviewed elsewhere.^249^ Together, further mechanistic insights into the cellular functions of lncRNAs in regulating neuroinflammation‐associated diseases will open new avenues for therapeutic intervention.

## 4 EPIGENETIC‐TARGETED THERAPY FOR INFLAMMATION‐RELATED DISEASES

With the continuous development of epigenetics, research into epigenetic‐related drugs has made substantial progress. At present, numerous small‐molecule inhibitors or activators involving epigenetic regulation pathways are being developed for clinical therapy. The target of epigenetic drugs mainly includes DNMTs, DNA demethylases, HDACs, HATs, histone methyltransferases (HMTs), histone lysine demethylases (KDMs), and others.^255^ Beside these common epigenetic regulators, miRNAs and lncRNAs are also involved in the regulation of many inflammatory diseases pathways. In recent years, they have also been used as therapeutic targets and diagnostic biomarkers for inflammation‐related diseases. The origin and development of inflammation‐related diseases are mainly attributed to excessive and persistent responses to self‐antigens, and they are also characterized by excessive inflammation.^256^ Thus, different inhibitors are used to treat inflammatory diseases caused by epigenetic modifications occurring during the immune response. Unlike gene therapy causing an increased risk of genetic mutations, targeting epigenetic regulators will be a secure and promising strategy for inflammatory diseases based on reversible process of epigenetic modification.

### 4.1 Application of DNA methylation in the treatment of inflammation‐related diseases

DNMTs and DNA demethylases coordinate regulation of the genome's methylation status.^257^ Thus, targeting DNMTs will be a potential therapeutic strategy to treat inflammatory diseases. Based on this principle, the molecule DNMT inhibitors (DNMTis) (Figure 4), commonly known as demethylating drugs, was designed.^258^ Currently, DNMTis are the primary treatment options for myelodysplastic syndromes (MDS) and acute myeloid leukemias (AML). Both azacitidine and decitabine were the first discovered DNMTis,^259^ and they were approved by the FDA for the treatment of chronic myelomonocytic leukemia and a range of other types of malignancies^118^ (Table 1). These nucleoside analogs inhibit DNA synthesis by binding to DNA during the S phase, forming irreversible complex with DNMTs during this phase. As a result, DNMTs are degrade with DNA demethylation, and silent related tumor suppressor genes are restored to expression.^255^, ^258^, ^260^ Recent studies have reviewed the antitumor effect of epigenetic drugs in various diseases.^255^, ^257^, ^259^, ^261^, ^262^, ^263^ And antitumor drugs also play an important role in treating chronic inflammation‐related diseases, including autoimmune disorders and ND. For example, azacitidine and decitabine extensively inhibit DNA methylation in macrophages, resulting in the inhibition of inflammation and IFN responses, and turn leading to a certain therapeutic effect on inflammatory diseases such as RA and COVID‐19. An animal model of RA showed that azacitidine significantly reduced the release of proinflammatory cytokines, such as IL‐6 and TNF‐α.^264^, ^265^ Meanwhile, Fu et al.^266^ showed that azicitidine induced IL‐10 production in RA PBMCs, suggesting that demethylation of the IL‐10 promoter by azacitidine could increase immune response.^264^ Additionally, Zhao et al.^267^ found that the expression of tumor necrosis factor receptor‐associated factor 1 and connective tissue growth factor significantly increased in osteoarthritis and RA chondrocytes. Rheumatoid synovium predominantly contains leukocytes, such as B cells, T cells, and phagocytes. However, decitabine treatment may lead to decreasing DNMT1 and EZH2 expression, while overexpression of DNMT1 and EZH2 leads to depletion of B cells and prevents the production of macrophages. This suggests that decitabine might be used to treat RA.^268^, ^269^ On the other hand, IL‐6/STAT3‐mediated downregulation of suppressor of cytokine signaling transduction 3 (SOCS3) could be reversed by decitabine, which could prevent hypermethylation of the SOCS3 promoter and increase the transcription of SOCS3.^270^ Therefore, decitabine is involved in the regulation of inflammatory response. A virus‐like SARS‐CoV‐2 infects cells by activating ACE2 and neurofibromin‐1 (NRP1) receptors and initiates S proteins by using a serine protease called TMPRSS2. As a result, inhibiting protease activity or binding to ACE2 and NRP1 receptors have been proven to be effective methods of blocking viral infectivity.^271^ Therefore, DNMT1 inhibitors including azacitidine and decitabine (Figure 4), can be repurposed to cure SARS‐CoV‐2 infection.^272^ Notably, decitabine has recently been reported to be in clinical trials for treatment of COVID‐19 (CTI: NCT04482621).^273^

**TABLE 1:** Important epigenetic modification inhibitors involved in inflammation‐related diseases

Modified types	Epigenetic drug name	Epigenetic effect	Status	Diseases
DNA methylation	Azacitidine	DNMT inhibitor	Approval in 2004	Cancer,^255^, ^259^ RA,^265^, ^266^ COVID‐19^272^
	Decitabine	DNMT1, DNMT3A inhibitor	Approval in 2006	Cancer,^255^, ^259^ RA,^267^ COVID‐19^272^, ^273^
	Zebularine	DNMT1 inhibitor	Clinical trial phase II	Cancer,^263^ AD^274^
	Guadecitabine	DNMT inhibitor	Clinical trial phase III	Cancer^257^, ^263^
	TdCyd	DNMT1 inhibitor	Clinical trial phase I	Cancer^259^
	FdCyd	DNMT1 inhibitor	Clinical trial phase I	Cancer^259^
	SGI‐1027	DNMT1, DNMT3A, DNMT3B inhibitor	Preclinical trial	Cancer^259^
	Nanaomycin A	DNMT3B inhibitor	Preclinical trial	Cancer^275^
	MG98	DNMT1 inhibitor	Clinical trial phase I	Cancer^276^
	RG108	DNMT1 inhibitor	Preclinical trial	Cancer^257^
	Resveratrol	Natural DNMT inhibitor	Preclinical trial	ND^277^
	Hydralazine	DNMT inhibitor	Clinical trial phase III	Cancer,^263^ RA,^278^ SLE^279^
Histone deacetylase	Vorinostat (SAHA)	HDAC1/2 inhibitor	Approval in 2006	Cancer,^261^ ND,^280^, ^281^ COVID‐19^273^
	Romidepsin	HDAC inhibitor	Approval in 2009	Cancer,^255^ RA^282^
	Belinostat	HDAC inhibitor	Approval in 2014	Cancer^283^
	Panobinostat	HDAC inhibitor	Approval in 2015	Cancer,^283^ COVID‐19^273^
	Valproic acid	HDAC2 inhibitor	Clinical trial phase I	Cancer,^284^ COVID‐19,^285^ AD,^256^ ND^286^
	RGFP966	HDAC3 inhibitor	Preclinical trial	Cancer,^287^ ND^288^
	Tubastatin A	HDAC6 inhibitor	Preclinical trial	Cancer,^289^ RA,^290^ COVID‐19,^291^ AD^256^
	WK2‐16	HDAC8 inhibitor	Preclinical trial	ND^292^
Histone methylation	Tazemetostat	EZH2 inhibitor	Approval in 2020	Cancer^293^
	GSK126	EZH2 inhibitor	Clinical trial phase I	Cancer^262^
	EPZ004777	DOT1L competitive inhibitor	Preclinical trial	Cancer^294^
	Chalcone	LSD1 inhibitor	Clinical trial phase II	Cancer, ND^295^
	T‐448	LSD1 inhibitor	Preclinical trial	ND^296^
	TAK‐418	LSD1 inhibitor	Clinical trial phase I	ND^297^

Abbreviations: AD, Alzheimer's disease; COVID‐19, coronavirus disease 2019; ND, neurodegenerative diseases; RA, rheumatoid arthritis; SLE, systemic lupus erythematosus.

With the deepening of clinical research, these nucleotide analogs exhibit many threats, including unstability chemically and metabolically, lack of specificity, and significant toxic side effects. Based on this phenomenon, more stable and less‐toxic cytidine analogs and their prodrugs: second‐generation DNMTis are designed and used for the treatment of inflammatory diseases. The second‐generation DNMTis comprises zebularine^259^, ^263^ and SGI‐110 (guadecitabine)^263^, ^298^ (Table 1). Xue et al.^274^ demonstrated that zebularine and IFN‐γ had similar function in inducing a immunosuppressive enzyme called indoleamine 2,3‐dioxygenase 1 (IDO1) which play a critical role in cell death. Both IFN‐α and zebularine interact synergistically to enhance IDO1 expression, so as do several other IFN‐α‐responsive transcription factors, such as STAT1 and IRF1. Therefore, zebularine may be an important immunosuppressant for the treatment of chronic autoimmune diseases. In addition, guadecitabine (SGI‐110) has been approved for clinical trials for the treatment of MDS and AML in phase II. A second‐generation hypomethylated compound combines decitabine with deoxyguanosine through phosphoric acid A diester‐linked dinucleotide, showing better therapeutic effects.^257^, ^298^

Additionally, analogs of non‐nucleosides have been discovered over the last few decades to resolve the poor bioavailability, chemical instability under physiological conditions, and lack of selectivity of nucleoside analogs. SGI‐1027 is a quinoline‐based compound with antioxidant activity against DNMT1 (Figure 4), DNMT3A, and DNMT3B^259^, ^299^ (Table 1). The first selective inhibitor of DNMT3B, nanaomycin A, can induce genomic demethylation in cells and bind to amino acid residues involved in DNA methylation, making it incapable of participating in the normal methylation of DNA^275^ (Table 1). In hepatoma cells, DNMT3B activation could increase octamer‐binding transcription factor 4 expression through the IL‐6/STAT3 pathway, as demonstrated by Lai et al.^300^, ^301^ Short‐chain oligodeoxynucleotides like MG98 prevent the translation of DNMT1 mRNA by binding to the 3ʹ‐UTR (Figure 4 and Table 1). Studies have shown that some patients with metastatic renal cell carcinoma achieve partial remission or stable disease after therapy with MG98 and type I IFN. This suggests that MG98 is safe and effective.^276^ N‐phthaloyl‐l‐tryptophan (RG108) (Figure 4), a DNMT1 inhibitor,^257^ targets DNMT1 for SAM cofactor binding. They directly bind to the catalytic region of DNMT rather than DNA. However, they are less used in other chronic inflammatory diseases. Grinan‐Ferre et al.^277^ comprehensively over‐viewed the main apparent mechanism of resveratrol (RV) in different inflammatory regulatory pathways, such as JAK/STAT and NF‐κB, revealing the role of RV in AD and PD. Kinsen et al. reported pleiotropic neuroprotection in ND. The above‐mentioned non‐nucleoside DNMTis have not yet approved for the clinical therapy, so new DNMTis which are selective, non‐nucleoside, and non‐toxic are still in their infancy. Autoimmune diseases are driven by epigenetic mechanisms that regulate B‐cell function. Studies have shown that hydralazine and procainamide, among other DNMTis, predispose patients to drug‐induced lupus erythematosus.^302^ Mazari et al.^303^ showed that B cells treated with hydralazine were passively transferred into syngeneic mice, and autoantibodies were detected more frequently because receptor editing and B‐cell tolerance were disrupted, demonstrating a causal relationship between loss of DNA methylation in B cells and the development of autoimmunity.

### 4.2 Application of histone modification in the treatment of inflammation‐related diseases

Therapeutic targeting of chromatin modifiers involved in the regulation of specific modulators of innate immunity and their specific inhibitors and activators will be a potential therapeutic strategy for inflammation‐related diseases. Research into the multifaceted effects of histone modification inhibitors has long positioned these drugs as promising drug candidates for the treatment of cancer, autoimmunity, neurodegenerative, and infectious diseases.

#### 4.2.1 Targeting histone acetylation

Based on extensive research targeting epigenetic regulators, HDAC inhibitors have aroused wide attention. HDAC inhibitors enhance the level of acetylation modification of histone or nonhistone lysine residues by inhibiting the activity of HDACs, controlling the tightness of DNA wrapping around histones, and making DNA wrap around histones more tightly, which leads to this DNA not being easily accessible to gene transcription factors (Figure 6). HDAC inhibitors based on the molecular structures are divided into five classes: hydroxamic acids, short‐chain fatty acids, benzamides, cyclic tetrapeptides, and sirtuin inhibitors.^304^

The FDA has also approved some HDAC inhibitors for clinical therapy. The first HDAC inhibitors approved for use in treating cutaneous T cell lymphoma (CTCL) were vorinostat (SAHA) and romidepsin^255^ (Table 1). SAHA has been proven to have good curative effects in liver cancer, cervical cancer, ovarian cancer, etc., in 2006 and is currently the third‐line treatment option for CTCL patients.^261^ This confirmed that SAHA had better oral absorption and lower cytotoxicity. Romidepsin was FDA‐approved as the second inhibitor of HDAC in 2009 (Table 1). Subsequently, a variety of therapies have been approved for relapsed and refractory peripheral T‐cell lymphoma (PTCL) or multiple myeloma, including belinostat, panobinostat, and chidamine.^283^ The studies of HDAC inhibitors in tumors have been reviewed,^255^, ^305^, ^306^ and after their introduction into cancer therapy, the application of HDAC inhibitors in other diseases has also drawn attention. A study reported by Dai et al.^307^ provided a systematic overview of the roles and mechanisms of HDACs in neuroinflammation, as well as the corresponding roles of HDAC inhibitors in the treatment of neurological disorders. NF‐κB, JAK/SATA, and TLR/MyD88 signaling pathways have been proved to play important roles on reducing neuroinflammation in the hippocampus by using SAHA,^281^ which inhibits microglial M1 polarization and attenuates HDAC1/2‐dependent neuroinflammation.^280^ Various cell lines (HUVEC, Caco‐2,HK‐2, Huh‐7, and BEAS‐2B) were used to analyze the effect of the HDAC2 inhibitor valproic acid (VPA) and other HDAC inhibitors toward ACE2 and NRP1 receptors (Table 1). The results showed that SARS‐CoV‐2 was less infectious when use VPA to downregulated ACE2 and NRP1.^271^, ^285^ Conversely, in human clinical trials, VPA appeared to worsen behavioral symptoms in Alzheimer disease patients compared with placebo patients.^308^ RGFP966, a selective inhibition of HDAC3, attenuates NF‐kB transcriptional activity and exhibits anti‐inflammatory effects.^288^, ^309^ Additionally, the HDAC2 inhibitor VPA and the HDAC3 inhibitor RGFP966 are reported to reduce neuropathic pain because of spinal cord nerve ligation and cerebral ischemia. In a recent study, Guan et al. demonstrated a correlation among HDAC2 and reduced synapse number and memory formation. And chronic treatment with SAHA restores memory impairment and reduces synapse number in HDAC2‐overexpressing mice.^310^ The inhibition of HDAC3 promoted STAT1 and p65 acetylation. Additionally, the inhibition of NF‐κB signaling could promote the polarization of MG to M2 and inhibit the release of inflammatory cytokines.^311^ Inflammation can be inhibited by the specific HDAC6 inhibitor tubastatin A^312^, ^313^ (Table 1). The HDAC6 inhibitor tubastatin A or the HDAC inhibitor trichostatin A (TSA) concomitantly could enhance the production of Treg and IL‐10, as well as FOXP3 expression, and also could suppress neuroinflammation in the CNS.^314^ Lin et al.^292^ found that a HDAC8‐specific inhibitor, WK2‐16, might inhibit glial cell activation via STAT1/3 and AKT signaling pathways, thereby protecting the CNS from inflammation (Table 1). Interestingly, there has been evidenced that HDAC inhibitors could reduce inflammation in several mouse models. In the animal model of RA, the HDAC inhibitor TSA inhibits the production of proinflammatory cytokines derived from inflamed synovial macrophages.^290^ A combination of TSA and MI192 inhibits IL‐6 production in PBMCs stimulated by LPS^315^ (Table 1). Similarly, romidepsin (FK228) and MPT0G009 inhibit synovial fibroblast proliferation^282^, ^316^ (Table 1). A single‐dose combination of azacitidine and TSA has been found to significantly reduce pulmonary vascular hyperpermeability and inflammatory lung injury after the onset of ALI. Additionally, combining treatment with azacitidine and TSA promotes the formation of anti‐inflammatory M2 macrophages in ALI patients.^291^, ^317^

#### 4.2.2 Targeting histone methylation

Studies have shown that histone methylations have undergone dramatic changes in inflammation‐related diseases including tumors,^318^ neurological diseases,^319^ autoimmune diseases,^135^ and COVID‐19^273^ targeted histones. As a result, methylation of histones will offer opportunities for curing these diseases. However, there are fewer drugs that targeted histone methylation approved by the FDA currently (Figure 6). Only small‐molecule inhibitors targeting enhancer of zeste homolog 2 (EZH2) and disruptor of telomeric silencing 1‐like (DOT1L) have entered clinical development. In 2020, the FDA approved for the first time (and to date only) the histone lysine methyltransferase (KMT) EZH2 inhibitor tazemetostat for epithelioid sarcoma and subsequent follicular lymphoma^293^ (Table 1). The proliferation of abnormal cells is linked to EZH2 mutations or abnormal activation. EZH2 inhibitors can reduce histone H3 lysine 27 (H3K27) methylation, and inhibit tumor growth.^320^ GSK126 effectively inhibits the activity of EZH2 methyltransferase by competing with SAM, resulting in an increase in cells derived from myeloid tissues that suppress myeloid proliferation and a decrease in CD4^+^ and IFN‐γ^+^CD8^+^ T cells involved in antitumor immunity.^321^ Following this, EPZ005687, EPZ‐6438, CPI‐1205T, and other EZH2 inhibitors have been currently developed and entered in phase I or phase II clinical trials (Table 1). Histone H3 lysine 79 (H3K79) methylation can be catalyzed by DOT1L and transferred methyl groups to lysine residues in the substrate.^322^ The first SAM‐competitive inhibitor of DOT1, EPZ004777, is effective against leukemia (MLL) and prolongs survival in MLL mice^318^ (Table 1). EPZ‐5676, a derivative of EPZ004777 (Figure 6), is another potent DOT1L inhibitor.^323^ Scheer et al.^324^ showed that the DOT1L gene was a key regulator of T‐cell development, and inheriting DOT1L deficiency reduced CD4^+^ and CD8^+^ T‐cell H3K79me2 levels. Based on the findings of Kealy et al.,^325^ a comprehensive understanding of DOT1L function in B and T cells is provided, extending our knowledge of lymphocyte gene expression. EZH2 and DOT1L serve as powerful immune response regulators, suggesting their small‐molecule inhibitors using to treat inflammatory conditions effectively.

With the continuous development of epigenetic mechanisms and experimental techniques, various inhibitors of histone methyltransferases and histone demethylases have been discovered.^326^ The inhibitor of lysine‐specific demethylase 1 (LSD1/KDM1A), a FAD‐dependent demethylase of H3K4 and H3K9, has been the only small‐molecule inhibitor of histone demethylase for clinical therapy.^327^ And the current status of its clinical inhibitor development is reviewed in Ref.^328^ The LSD1 inhibitor chalcone (a,b‐unsaturated aromatic ketone) has clinical potential against a variety of diseases due to its broad pharmacological activities, including antioxidant, antiulcer, antiviral, anticancer, antibacterial, antiangiogenic, and anti‐inflammatory activities.^329^ T‐448 induces LSD1 demethylase activity in the brain, and improve learning ability in mice with little hematologic toxicity, suggesting that it may be a promising treatment for neurodevelopmental disorders^296^ (Table 1). TAK‐418, a clinical drug candidate, was identified (CTI: NCT03228433, NCT04202497, NCT03501069) (Table 1). In mouse models of neurodevelopmental disorders, TAK‐418 treatment mice normalized dysregulated mRNA expression by restoring homeostatic regulation of global gene expression.^297^ These results suggest that neurodevelopmental disorders may be treated with TAK‐418 by inhibiting LSD1 enzymatic activity, thereby stabilizing aberrant gene expression through epigenetic regulation to restore global pathological gene expression. Kohanbash et al. showed that mutations in IDH1 and IDH2 decreased STAT1 expression in glioma cells, causing by hypermethylation at the STAT1 promoter. Therefore, IDH1 and IDH2 mutations lead to a reduction of effector molecules associated with type 1 tumors and chemokines such as CXCL10 in CD8^+^ T cells.^330^, ^331^ Since IDH mutations cause histone hypermethylation throughout the genome by broadly inhibiting the activity of HDMs, targeting the metabolic enzyme IDH mutation as a target for tumors and inflammation‐related diseases has been a promising treatment. In addition, SETDB1, PRMT5, EZH2, JARID1C, etc., may be potential therapeutic targets for ND treatment.^319^ Inactivation of Tet2 in mice completely prevented previous inflammation‐induced loss of substantia nigra dopaminergic neurons. Loss of Tet2 also attenuated transcriptional immune responses to inflammation triggers. Therefore, the extensive epigenetic dysregulation of enhancers observed in PD neurons may be partly mediated by the increased expression of TET2. So it may be possible to treat PD by reducing TET2 activity in vivo.^332^

### 4.3 Application of RNA modification in the treatment of inflammation‐related diseases

The m6A RNA modifications are the most common RNA modifications in eukaryotes, and involved in multiple aspects of RNA metabolism such as splicing, stability, structure, translation, and export.^333^ Previous studies have shown that the physiological and pathological activities of innate immune cells are regulated by m6A, and also demonstrated that m6A modifications are involved in mediating the mechanisms of innate and adaptive immune response in inflammatory‐related diseases such as HIV and SARS‐CoV‐2. During infection, RNA regulation by m6A also promote an antiviral response. Besides, the dysregulation RNA methylation is associated with many inflammation‐related diseases, such as neurodevelopmental disorders and ND.^334^ Therefore, inhibiting of RNA methylation is expected to improve the treatment of chronic inflammatory diseases, cancer, and antiviral infections. Li et al.^335^ analyzed the molecular alterations and gene expression of m6A modulators (writer, eraser, and reader) in diseases, providing prospective insights into the further development of therapeutic targets. Unfortunately, the current clinical research on m6A modification is still in the stage of animal models, and only a few small‐molecule activators or inhibitors are effective. Therefore, the design of molecular drugs targeting m6A deserves exploration.

METTL3 and METTL14, m6A writers, play an important functions in tumors and the immune system and serve as potential therapeutic targets. Overexpression of *METTL3* and *METTL14* inhibits the growth of cancer cells. For example, Cui et al.^336^ found that mice transplanted with *Mettl3*‐ or *Mettl4*‐knockout glioblastoma stem cells (GSCs) had larger tumors compared to control group. After *Mettl3* overexpression or *Fto* inhibition, GSC growth and self‐renewal are promoted and mice survived longer.^336^ But in rheumatic arthritis, the results showed that METTL3 triggers fibroblast‐like synoviocyte activation and inflammation via the NF‐κB pathway, thereby speeding up the onset and progression of rheumatic arthritis.^337^ The expression of m6A regulators, including METTL3 and METTL14, is downregulated in SLE patients, thus METTL3 and METTL14 serve as a potential biomarkers for assessing risk and disease activity.^338^ According to the research on APP/PS1 transgenic AD mouse models, it showed that elevated expression levels of m6A in the mouse cortex and hippocampal regions, as well as the expression of *Mettl3* and *Fto*.^339^ Conditional ablation of METTL14 in vivo results in reduced oligodendrocyte numbers and decreased myelination of the CNS. Further evidence suggested METTL14 was associated with neurological disorders resulting from disruption of postmitotic oligodendrocyte maturation.^340^ As a regulator of innate and adaptive immunity, METTL3 regulates macrophages and DCs in the host,^341^ METTL3 overexpression significantly inhibits the LPS‐induced macrophage inflammatory response through the NF‐κB pathway.^342^ By eliminating METTL3, T cells remain in the naïve stage for a longer time by methylating m6A to target IL‐7/STAT5/SOCS.^343^ METTL3 and METTL14 have different functions in various inflammatory‐related diseases, thereby targeting METTL3 and METTL14 could contribute to the treatment of inflammatory diseases. Although few small‐molecule inhibitors of MELL3 have been reported, but drugs in development have been published recently and are approaching for phase I clinical trials.^344^

FTO and ALKBH5, two “erasers” of m6A, also play an important role in regulating inflammatory diseases. As reviewed by Garbo et al.,^344^ some inhibitors targeting these two demethylases have been developed to treat with cancer, but also have benefits in inflammatory‐related diseases. The FTO inhibitors, FB23 and FB23‐2, selectively inhibit the m6A demethylase activity of FTO, thereby significantly inhibiting the proliferation of AML cells and promoting apoptosis.^345^ Additionally, two compounds named CS1 and CS2 have potent antitumor effects in multiple cancer types by directly binding to the FTO protein and blocking its catalysis.^346^ Recently, MO‐I‐500 was identified as a selective inhibitor of FTO that inhibits the proliferation of triple‐negative breast cancer cells.^347^ It has been demonstrated that meclofenamic acid and N‐(5‐chloro‐2,4‐dihydroxyphenyl)‐1‐phenylcyclobutanecarboxamide which are nonsteroidal anti‐inflammatory drugs, competing with FTO for binding to m6A‐containing sequences to inhibit its activity.^348^, ^349^ A high level of FTO was found in human melanoma, and knockdown of *FTO* increased m6A methylation in PD‐1, CXCR4, and SOX10, causing increased RNA decay through the m6A reader YTHDF2. Consequently, *FTO* depletion enhanced the response of mouse melanoma cells to IFN‐γ and anti‐PD‐1 therapies.^350^ Recent studies have demonstrated the therapeutic potential of dCas13b‐ALKBH5 fusion proteins which targeted mRNA demethylation.^351^ During viral infection, the RNA helicase DDX46 binds more strongly to transcripts, which encode MAVS, TRAF3, and TRAF6, and other antiviral protein, resulting in the recruitment of ALKBH5 to demethylate these transcripts.^352^ The number of proliferating cells was significantly increased in the cerebellum of *Alkbh5*‐deficient mice, but mature neurons were reduced,^353^ suggesting that *Alkbh5* deficiency affects the proliferation and differentiation of neuronal progenitors.

m6A “readers” which recognize m6A modifications, are also important for the regulation of inflammatory diseases. The initiation of T‐cell immune responses is enhanced in *Ythdf1*‐deficient mice, with stronger activation of antitumor CD8^+^ T cell.^354^ Furthermore, a recent study by He et al. showed that a YTHDF1‐dependent mechanism regulates durable neoantigen‐specific immunity, and antigen‐specific CD8^+^ T‐cell responses against tumors were observed in *Ythdf1*‐deficient mice. The efficacy of PD‐L1 checkpoint blockade was also enhanced.^355^ In addition, YTHDF2 sequesters m6A‐circRNA, which is essential for suppressing innate immunity.^356^ It has been discovered that PBMCs of RA patients expressed less ALKBH5, FTO, and YTHDF2,^357^ which also revealed the potential of these proteins for assessing RA risk and progression. According to previous studies, ribavirin bound to m7G to block translation of mRNA, and had therapeutic value in the treatment of tumors by competing with eIF4E. With the progress of RNA methylation research, drugs of ribavirin paired with m7G‐modifying are potentially used to treat inflammation‐related diseases.^358^

### 4.4 Application of noncoding RNA in the treatment of inflammation‐related diseases

Regulatory ncRNAs, including small noncoding RNAs, lncRNAs, and circRNAs, have gradually been discovered in the past few years for their mechanisms of action in inflammatory diseases. As therapeutic targets and promising biomarkers for inflammatory diseases, they have aroused extensive attention. RNA‐based drugs are designed and used in treating inflammation‐related diseases. miRNAs negatively regulate mRNA expression, thereby interfering with posttranscriptional regulation in various diseases. Likewise, lncRNAs are involved in cellular processes by affecting epigenetic, posttranscriptional, and translational pathways.^190^ Oligonucleotides complementary to miRNAs can block their activity,^359^ while dsRNAs or chemically modified ssRNAs mimicking miRNAs^360^ can trigger enhanced activity^361^ for the treatment of different human diseases. miRNA inhibitors and mimetics are currently being developed and tested in clinical trials aiming at multiple targets. Single‐stranded oligonucleotides called antisense oligonucleotides inhibit translation of target genes with their interaction with RNA. Their interaction is based on its DNA sequence complements with the target RNA transcript, causing the DNA degradation and preventing protein synthesis.^362^

Currently, 40 varieties of SARS‐CoV‐2 miRNAs and their regulatory targets have been identified.^363^ There has been extensive investigation into miRNAs which act as potential diagnostic, prognostic, and therapeutic markers for AD due to their involvement in a variety of brain signaling pathways.^364^ Atlante et al.^273^ summarized some epigenetic targets and interventions that may be useful in the treatment of coronavirus infection. miRNAs are ssRNAs encoded by endogenous genes that target and decay mRNA transcripts posttranscriptionally. The serum concentration of miRNA‐5196 can function as a biomarker to predict positive treatment outcomes in TNF‐α‐treated patients with RA and ankylosing spondylitis. Visual inspection is the most common method of diagnosing RA.^365^ Blood tests are used as an adjunct to comprehensive analysis. The recent SARS‐CoV‐2 pandemic has caused unprecedented progress in RNA vaccine development.^366^ CircRNAs play a crucial role in immune responses based on their diverse modes of action. By forming intramolecular duplexes and interacting with DNA, other RNAs, and RBPs, they are circular and contain several structural features. These new RNA therapeutics have a lot of promise and are versatile. Furthermore, rapidly growing data clearly indicate that nearly 40% of lncRNAs are specific to the nervous system. Consequently, these dysregulated lncRNAs may be involved in chronic neuropathic pain occurrence, development, and progression.^367^ Humans and mice innate antiviral and antibacterial immune responses to lncRNAs.^368^ Disorders of the expression of many lncRNAs have been detected in AD,^369^, ^370^ which its pathological processes comprises Aβ deposition, hyper‐phosphorylation of Tau protein, oxidative stress, neuroinflammation, mitochondrial dysfunction, and the regulation of autophagy. Furthermore, regarding the mechanism of AD neuroinflammation, a PC12 AD model and a primary neuronal AD model showed that MALAT1 downregulates IL‐6 and TNF‐α expression, but enhances IL‐10 expression. Based on these results, MALAT1 plays an important role in the development of AD by regulating inflammation. It has also been shown that MALAT1 inhibits miRNAs involved in inflammation, including miR‐125b and miR155. MiRNA targets are involved in a number of signaling pathways, including NF‐κB signaling pathway, the JAK signaling and activator of transcription (STAT) pathway, p38 signaling pathway, and JNK pathway, thereby inhibiting the neural pathways of AD inflammation.^371^ The SNHG1 RNA functions as a ceRNA of miR‐7, regulating the expression of NLRP3 and activating the NLRP3 inflammasome.^253^ The GAS5 regulated the NLRP3 pathway by sponging miR223‐3p in PD, which may promote microglial inflammation.^372^

## 5 CONCLUDING REMARKS AND PERSPECTIVES

Chronic inflammation causes various inflammation‐related diseases during the immune response of cells and the body. Epigenetic modifications have been accordingly changed with the occurrence and development of inflammation reaction. In turn, epigenetic alterations intercede inflammatory actions. Therefore, it is expected to reverse chronic inflammatory environment in the body via targeting these epigenetic regulators. Although clinical epigenetic drugs are still in its nascent stage, several epigenetic drugs have been approved, and a series of epigenetic marks exist or are under development for disease diagnosis, treatment response, and prognosis. Tumor therapy is currently the focus of clinical epigenetics, but numerous clinical trials and preclinical studies are also being conducted in other areas of medicine beyond cancer (ND, autoimmune diseases, cardiovascular diseases, etc.). Together, targeting epigenetic regulators would be an effective strategies for inflammatory diseases treatment by using predictive biomarkers or novel screening methods. Compared with traditional chemotherapeutics, epigenetic drugs show great advantages in the diversity and feasibility of biotherapeutics, but their clinical application hardly utilized. There are still many problems that are difficult to solve. We summarize as follows.

First, based on the complex regulation of epigenetic modification, this therapeutic approach may be limited by its lack of specificity. Currently, FDA‐approved drugs, located at multiple genomic loci, play an equal role in re‐expression of silenced genes, which potentially can reactivating nonsense sequences in the genome, but have no differential effect on their epigenetic marks. Second, interactions between epigenetic factors give rise to the “promiscuous” nature of specific epigenome‐modifying enzymes. Multiple PTMs of histones can target the same amino acid residue, resulting in competitive antagonism at these PTMs sites. Therefore, targeting inhibition or activation of one PTM may lead to changes in the other modifications, thus affecting other cell functions. As an example, targeting HDAC inhibitors, such as vorinostat and sodium butyrate, can also alter histone methylation and the binding of chromatin remodeling factors. In addition, HDAC inhibitors can also alter CpG methylation at specific promoters by affecting DNMT activity. One reason for this interaction is that epigenetic modifiers form complex with other chromatin‐associated proteins. As a result, interventions in one partner may have unforeseen consequences for other partners. Epigenetic drugs frequently suffer from these drawbacks, which are often accompanied by significant side effects. Third, the majority of epigenetic drugs have been designed to target DNA methylation, histone methylation, and histone acetylation. Although the role of some epigenetic modifications in inflammation‐related diseases has been elucidated, candidate drugs targeting in chromatin openness, RNA modification, and DNA modification remain unidentified. Therefore, targets for these modifications will be an underlying clinical strategy for inflammatory disease. Fourth, the use of epigenetic drugs may benefit some patients with inflammatory diseases, but liking cancer chemotherapy, not all patients can benefit from monotherapy. So far, the FDA and other regulatory agencies have rarely approved epigenetic drugs for advanced cancer and inflammation‐related diseases due to insufficient efficacy, except for a few types of solid tumors. Therefore, it is possible to improve the therapeutic antitumor efficacy of epigenetic drugs by combining with other drugs. As the field of immunology develops, it is believed that future consortium of therapeutic modalities such as epigenetic therapy and immunotherapy are rapidly emerging as new paradigms for the treatment of inflammation‐related diseases.

## CONFLICT OF INTEREST

The authors declare they have no conflicts of interest.

## AUTHOR CONTRIBUTIONS

S.Z., Y.M., L.Z., and J.H. conceived, edited, and wrote this manuscript. L.Q., H.W., D.S., B.Z., K.‐M.C., and J.H. revised this manuscript. All authors have read and approved the final manuscript.

## ETHICS STATEMENT

The author declare that ethics approval was not needed for this study.

## Data Availability

Not applicable.
